# Cerebellar Single‐Pulse TMS Differentially Affects Early and Late Error Processing in Reinforcement Learning

**DOI:** 10.1111/psyp.70178

**Published:** 2025-10-31

**Authors:** Dana M. Huvermann, Adam M. Berlijn, Stefan J. Groiss, Manfred Mittelstaedt, Alfons Schnitzler, Christian Bellebaum, Martina Minnerop, Dagmar Timmann, Jutta Peterburs

**Affiliations:** ^1^ Faculty of Mathematics and Natural Sciences Heinrich Heine University Dusseldorf Dusseldorf Germany; ^2^ Department of Neurology and Center for Translational and Behavioral Neurosciences (C‐TNBS), Essen University Hospital University of Duisburg‐Essen Essen Germany; ^3^ Institute of Clinical Neuroscience and Medical Psychology, Medical Faculty & University Hospital Düsseldorf Heinrich‐Heine University Düsseldorf Düsseldorf Germany; ^4^ Institute of Neuroscience and Medicine (INM‐1) Research Centre Jülich Jülich Germany; ^5^ Department of Neurology, Center for Movement Disorders and Neuromodulation, Medical Faculty Heinrich‐Heine University Düsseldorf Düsseldorf Germany; ^6^ Institute for Systems Medicine & Department of Human Medicine MSH Medical School Hamburg Hamburg Germany

**Keywords:** cerebellum, cognitive control, ERN, ERP, performance monitoring, reinforcement learning, single‐pulse transcranial magnetic stimulation

## Abstract

There is increasing evidence that the cerebellum contributes to feedback processing in reinforcement learning. As yet, it has not been investigated whether the cerebellum also contributes to error processing in reinforcement learning. Studies have shown, however, that the cerebellum is involved in the processing of response errors in non‐reinforcement learning contexts, for example, in response conflict tasks. In the present study, we aimed to extend these findings to the processing of response errors, which slowly emerges as a result of reinforcement learning. To this end, we inhibited the cerebellum via single‐pulse transcranial magnetic stimulation (spTMS) and recorded cerebral electroencephalography (EEG) measures associated with error processing. If input from the cerebellum is required for error processing, error‐correct differentiation should be decreased for cerebellar compared to vertex (control) stimulation. Cerebellar spTMS was applied and EEG was recorded while healthy adults performed a probabilistic feedback learning task. The error‐related negativity (ERN), a component in the response‐locked event‐related potential (ERP), was used as a measure of error processing. It reflects a rapidly detected mismatch between representations of the actual and the desired response and is typically larger for errors than correct responses. Error‐correct differentiation in the ERN was diminished for cerebellar compared to control TMS. However, increased error‐correct differentiation was found in a later ERP component, the error positivity (Pe), which is more strongly associated with error awareness. Cerebellar spTMS thus impaired fast error processing reflected in the ERN and facilitated later, conscious error processing reflected in the Pe. These findings provide causal evidence of cerebellar contributions to error processing within reinforcement learning.

## IMPACT STATEMENT

This study showed that single‐pulse TMS‐induced disruption of cerebellar function alters cerebral error processing as captured in the error‐related negativity (ERN). These results align with prior research on impaired error processing in patients with cerebellar damage during response inhibition tasks and extend this evidence toward reinforcement learning contexts. Our findings further add to a growing body of evidence relating the cerebellum to reinforcement learning, influencing both error and feedback processing.

## 1 Introduction

Understanding how organisms optimize their behavior in dynamic environments is crucial not only to improve learning processes but also to advance our understanding of disorders associated with maladaptive learning, such as addiction and depression (Gueguen et al. 2021; Chen et al. 2015). Reinforcement learning is a basic form of learning in which behavior is shaped by its consequences/outcomes, that is, rewards that reinforce and punishments that inhibit a specific behavior (Sutton and Barto 2018). Initially, in an unfamiliar context, information about actions and outcomes must be gathered on a trial‐and‐error basis. With learning, actions are then chosen based on their predicted outcomes. Learning success thus strongly depends on the accuracy of outcome predictions. While improving these predictions, the individual gets a better understanding of which action is correct and which is false. Ultimately, the individual is able to identify an error already at the stage of action execution, rather than having to wait for external feedback/the outcome. This shift from outcome‐level processing to response‐level processing underlying the distinction between right and wrong responses throughout the learning process could be shown in a reinforcement learning task by recording brain activity using electroencephalography (EEG; Eppinger et al. 2008; Bellebaum and Colosio 2014).

Processing of both actions/responses and outcomes has been predominantly linked to structures in the fore‐ and midbrain (Corlett et al. 2022). In EEG studies, error processing has been shown to emerge with learning/task progression when an understanding of correct and false responses has been developed (Eppinger et al. 2008; Bellebaum and Colosio 2014; Pietschmann et al. 2008). In later stages of a learning task, a more pronounced negative deflection in the response‐locked signal is typically found for errors relative to correct responses (Eppinger et al. 2008; Bellebaum and Colosio 2014; Pietschmann et al. 2008), that is, the error‐related negativity (ERN; Falkenstein et al. 1991; Gehring et al. 1993). The ERN has a frontocentral scalp distribution and typically peaks within 100 ms post‐response. Its origin lies primarily in the anterior cingulate cortex (ACC, Dehaene et al. 1994; Miltner et al. 2003; Iannaccone et al. 2015, but also see Herrmann et al. 2004) which has been associated with error processing (Hester et al. 2004). The ERN is followed by the more posterior error positivity (Pe, peaking 200–400 ms post‐response, Falkenstein et al. 1991; Wessel 2012). ERN and Pe have been proposed to be functionally distinct (Wessel 2012), with the ERN reflecting a fast‐paced mismatch between the actual and desired response (Coles et al. 2001; Nieuwenhuis et al. 2001), and the Pe reflecting more conscious error processing (Nieuwenhuis et al. 2001; Ridderinkhof et al. 2009). On the other hand, feedback processing, as reflected in the feedback‐related negativity (FRN), is typically found at early stages of reinforcement learning where participants strongly depend on external feedback to perform the task accurately (Eppinger et al. 2008; Bellebaum and Colosio 2014; Pietschmann et al. 2008). The FRN has been described as a functional equivalent of the ERN during feedback processing, as both seem to contribute toward an adjustment of behavior toward error correction (Gentsch et al. 2009). In addition, there seems to be a high overlap in topography and neural generators (Gentsch et al. 2009; Holroyd and Coles 2002; Potts et al. 2011).

Interestingly, recent studies in rodents (Kostadinov and Häusser 2022) and humans (Huvermann et al. 2025; Rustemeier et al. 2016; Berlijn et al. 2025) have provided evidence for a potentially supportive role of the cerebellum in feedback processing during reinforcement learning (Peterburs and Desmond 2016). The cerebellum is best known for predictive processes in the context of motor control (Popa and Ebner 2019) but in the last decades increasingly also for cognitive processes (Berlijn et al. 2024b; Sokolov et al. 2017). The cerebellum is thought to support both motor and cognitive function by predicting outcomes via internal forward models (Popa and Ebner 2019; Wolpert et al. 1998; Tanaka et al. 2020), connecting with a wide range of cerebral brain areas, including the ACC, in a closed‐loop fashion (Ramnani 2012; Schmahmann and Pandya 1997; Glickstein et al. 1985; Kruithof et al. 2023; Habas 2021; Bostan and Strick 2018). Cerebellar dysfunction might thus influence feedback processing as reflected in the FRN via maladaptive support of ACC function. Indeed, in recent studies (Huvermann et al. 2025; Berlijn et al. 2025), we found that cerebellar lesions, degeneration, and TMS disrupted feedback processing in the sense that the prediction error was not represented in the FRN.

These previous studies (Huvermann et al. 2025; Rustemeier et al. 2016; Berlijn et al. 2025) have focused on the role of the cerebellum at the outcome stage. However, prediction at the response stage (i.e., error processing), as described above, is also a prominent part of reinforcement learning. Cerebellar damage and disruption of cerebellar function by non‐invasive brain stimulation have already been associated with deficits in error processing in response conflict tasks (Peterburs et al. 2012, 2015; Berlijn et al. 2024a; Tunc et al. 2019). Specifically, differentiation between errors and correct responses in the ERN was consistently reduced for cerebellar dysfunction (Peterburs et al. 2012, 2015; Berlijn et al. 2024a, only on trend level in Tunc et al. 2019). For the Pe, findings are more heterogeneous, with most studies not finding effects of cerebellar dysfunction, except for one study in cerebellar post‐acute stroke which showed increased error‐correct differentiation that was interpreted as compensatory for deficient error processing in the ERN (Peterburs et al. 2012). Response conflict tasks, however, contain no feedback and can instead be performed based on the initial instructions. For example, in a flanker task, participants need to indicate the direction of a central arrow in the presence of flanking arrows. Predictions thus do not evolve slowly with learning as in reinforcement learning.

In summary, previous studies support a cerebellar role in outcome processing in reinforcement learning and error processing in response conflict tasks. This is consistent with the proposed role of the cerebellum in performance monitoring, that is, in functions which support adaptive behavior, to which both reinforcement learning and error processing contribute (Peterburs and Desmond 2016). Error and feedback processing are closely intertwined, and it seems conceivable that in reinforcement learning tasks, disrupted feedback processing (on which participants rely in particular early in the task) caused by cerebellar dysfunction leads to changes in error processing (which emerges later in the task, with learning from feedback). These changes may be similar to those found for error processing under cerebellar dysfunction in response conflict tasks (Peterburs et al. 2012, 2015; Berlijn et al. 2024a; Tunc et al. 2019).

In the current study, we aimed to examine if aberrant feedback processing in cerebellar dysfunction transfers to the response phase with learning progression in a reinforcement learning task. We disrupted cerebellar function by non‐invasive brain stimulation in young adults. Single‐pulse TMS (spTMS) excites the subjacent neuronal populations followed by a prolonged period of reduced activity (Romero et al. 2019), potentially leading to inhibition or facilitation depending on various factors including stimulation site and timing (Shirota and Ugawa 2024; Luber and Lisanby 2014). For cerebellar stimulation, an inhibitory effect of spTMS on cortical function is mostly assumed (Desmond et al. 2005; Schutter and van Honk 2006; Viñas‐Guasch et al. 2023, but also see Du et al. 2018, for a review see Fernandez et al. 2020). We analyzed data from a previous study by our group (Huvermann et al. 2025) which were collected in young, healthy adults who received cerebellar spTMS while performing a probabilistic feedback learning task with trial‐by‐trial feedback. Importantly, overall learning performance was not affected by the TMS, in theory enabling error processing as the task progresses and learning takes place (Eppinger et al. 2008; Bellebaum and Colosio 2014; Pietschmann et al. 2008). ERN and Pe were analyzed as EEG indices of error processing. In accordance with previous work in response conflict tasks (Peterburs et al. 2012, 2015; Berlijn et al. 2024a), we expected to see reduced or absent error‐correct differentiation in the ERN for cerebellar TMS (Iannaccone et al. 2015, but also see Berlijn et al. 2024b). We expected to see this effect more strongly later in the task when response‐outcome contingencies have been learnt and error processing is more pronounced (Eppinger et al. 2008; Bellebaum and Colosio 2014; Pietschmann et al. 2008). However, we did not expect to see distinct compensatory mechanisms indexed by an increased Pe as observed in cerebellar stroke patients (Peterburs et al. 2012) due to the immediate effect of spTMS. Two stimulation timings were used, to differentiate direct disruption of error processing (via post‐stimulus/pre‐response TMS) from indirect effects of disrupted feedback processing on error processing (pre‐feedback TMS) due to maladjusted predictive processes.

In line with the hypotheses, we found decreased error‐correct differentiation in the ERN for cerebellar TMS. In addition, error‐correct differentiation in the Pe was increased for cerebellar stimulation while behavioral performance was overall preserved.

## 2 Material and Methods

The present study was part of a larger investigation of cerebellar contributions to reinforcement learning and presents novel, follow‐up analyses of data reported previously by our group (Huvermann et al. 2025). There, we focused on outcome/feedback processing and thus did not analyze response‐locked ERPs. We performed two studies on reinforcement learning, one with cerebellar stroke patients and respective controls, the other with healthy adults using cerebellar (vs. vertex) spTMS. The present work is focused on the spTMS study, because older adults typically show only weak error‐correct differentiation in the response‐locked ERP in reinforcement learning (Eppinger et al. 2008; Pietschmann et al. 2008; Herbert et al. 2011). However, analogous analyses were also performed for data from the patient study and are provided in Supplementary Analysis S1.

### 2.1 Participants

Sample characteristics are detailed in Huvermann et al. (2025). Data from 24 healthy participants (7 men, 17 women; mean age 23.3 years, SD = 2.9 years, age range 19–30 years) entered the analyses. According to the Edinburgh Handedness Inventory (Oldfield 1971) scores, 20 participants were right‐handed, two left‐handed, and two ambidextrous.

All participants gave written informed consent prior to participation. The study was conducted in accordance with the ethical principles for medical research involving human subjects outlined in the Declaration of Helsinki and approved by the Ethics Committees at the Faculty of Medicine of Heinrich‐Heine‐University Düsseldorf (2018‐240_1) and the University Hospital Essen (18‐8477‐BO).

### 2.2 Procedure

**FIGURE 1:**
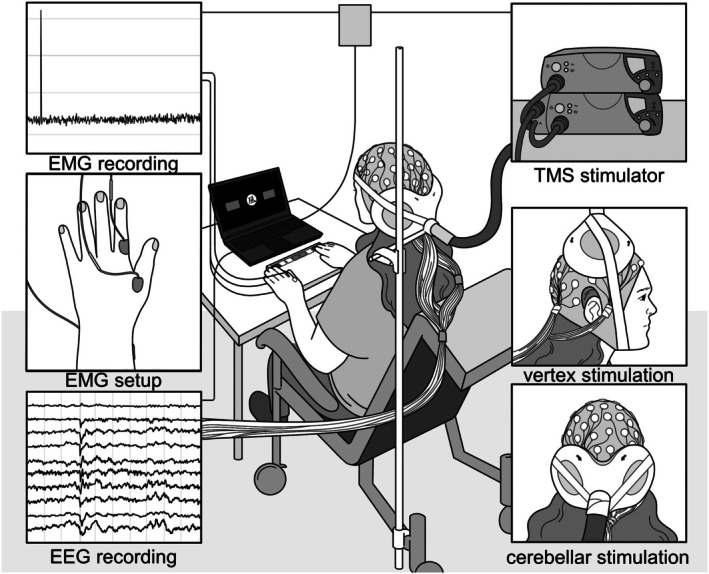
Experimental setup. Depending on the session, TMS was applied to either the left cerebellum (1 cm down and 3 cm to the left of the inion) or vertex using a double cone coil. EEG and EMG were recorded simultaneously. Reproduced from Huvermann et al. (2025) with permission.

Please see Huvermann et al. (2025) for a detailed description. In brief, cerebellar and vertex TMS took place in separate sessions at least 48 h apart to decrease repetition effects in the task. After EEG and EMG preparations and motor threshold estimation, the double cone TMS coil was positioned and secured to the participant's head (see Figure 1). Before and after the experimental task, an additional cognitive task was performed for which results are reported in Berlijn et al. (2024a).

**FIGURE 2:**
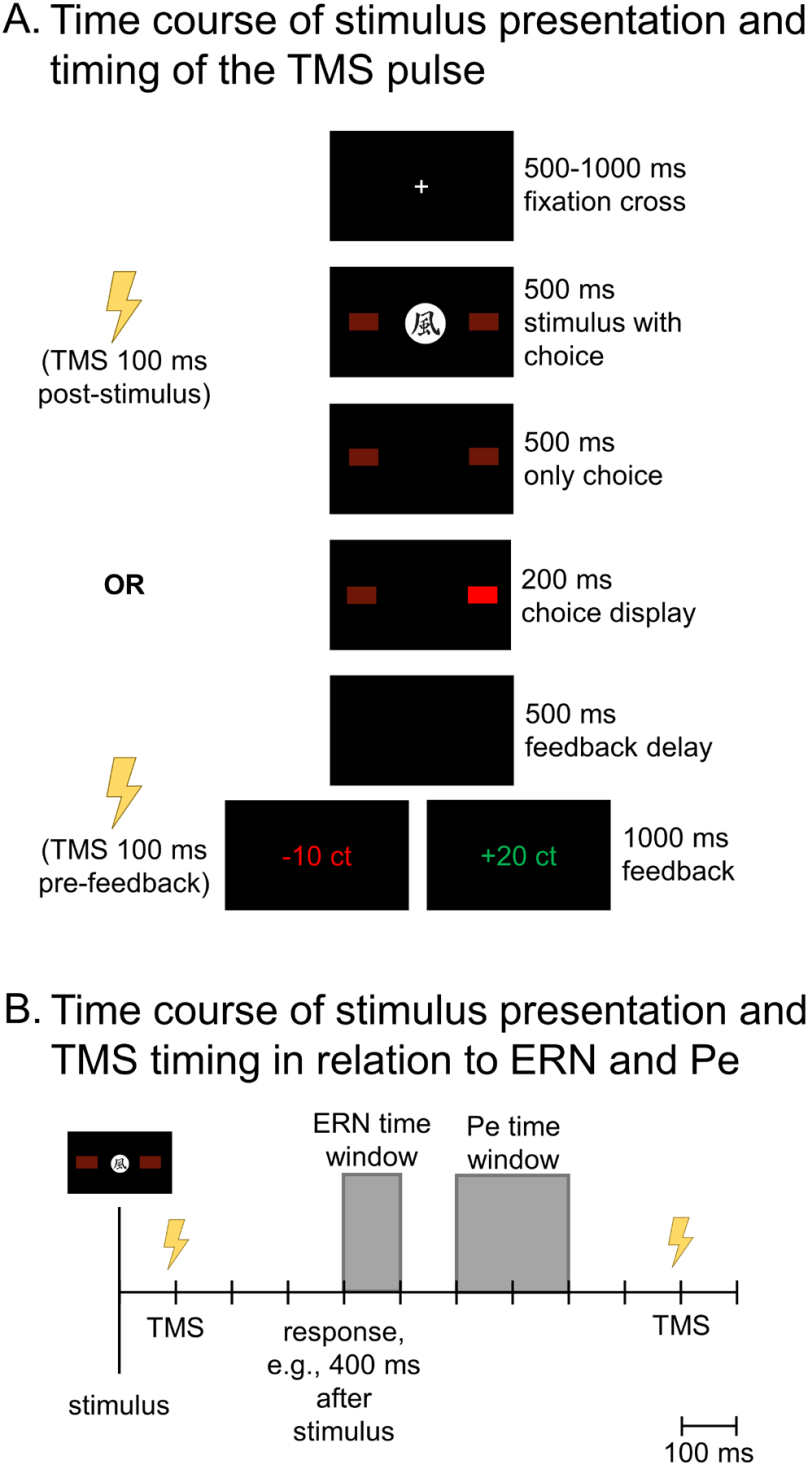
Time course in one trial in the experimental task. (A) Time course of stimulus presentation and timing of TMS pulses in one trial in the experimental task. First, a fixation cross was presented for 500–1000 ms. Subsequently, one of four stimuli was presented for 500 ms together with flanking rectangles representing the response options. Participants responded by pressing the left or right button on a response pad up until 500 ms after the stimulus was presented. The respective rectangle was highlighted for 200 ms. After 500 ms of blank screen, positive (“+20 ct”) or negative feedback (“−10 ct”) was presented for 1000 ms. Participants had to learn by trial and error which of the two options was more likely to result in positive/negative feedback, separately for each of the four stimuli. Feedback for two stimuli had an 80% contingency, and a 50% contingency for the other two. Stimulation in a particular trial was applied either 100 ms post‐stimulus or 100 ms pre‐feedback. The task consisted of 336 trials. (B) Time course of stimulus presentation and timing of the TMS pulse in relation to ERN and Pe time windows in one trial in the experimental task. ERN was quantified in the time window between 0 and 100 ms following the response while Pe was quantified in the time window between 200 and 400 ms following the response. Distance to the post‐stimulus TMS pulse thus differed and depended on response time in the respective trial, while the pre‐feedback TMS pulse always occurred after ERN and Pe. Note that the TMS pulse in a particular trial was given either post‐stimulus or pre‐feedback.

Participants completed a probabilistic feedback learning task (Eppinger et al. 2008; Bellebaum and Colosio 2014). Figure 2 illustrates the sequence and time course of stimulus presentation in each trial. The task consisted of 6 blocks of 56 trials, thus 336 trials in total. Each trial began with a fixation cross, followed by one of four stimuli (Chinese characters). Participants responded by pressing the left or right button on a response box within a response window of 1000 ms. Choices were highlighted on the screen, followed by a black screen before feedback was displayed, with “+20ct” in green font as positive feedback or “−10ct” in red font as negative feedback. Two stimuli were linked to random feedback (50% positive and 50% negative, independent of response), while the other two stimuli were linked to contingent feedback. Here, correct responses were followed by positive feedback in 80% of the cases and by negative feedback in 20% of the cases (vice versa for errors). Contingencies could thus be learnt. TMS was delivered 100 ms post‐stimulus for one stimulus and 100 ms pre‐feedback for the other.

**FIGURE 3:**
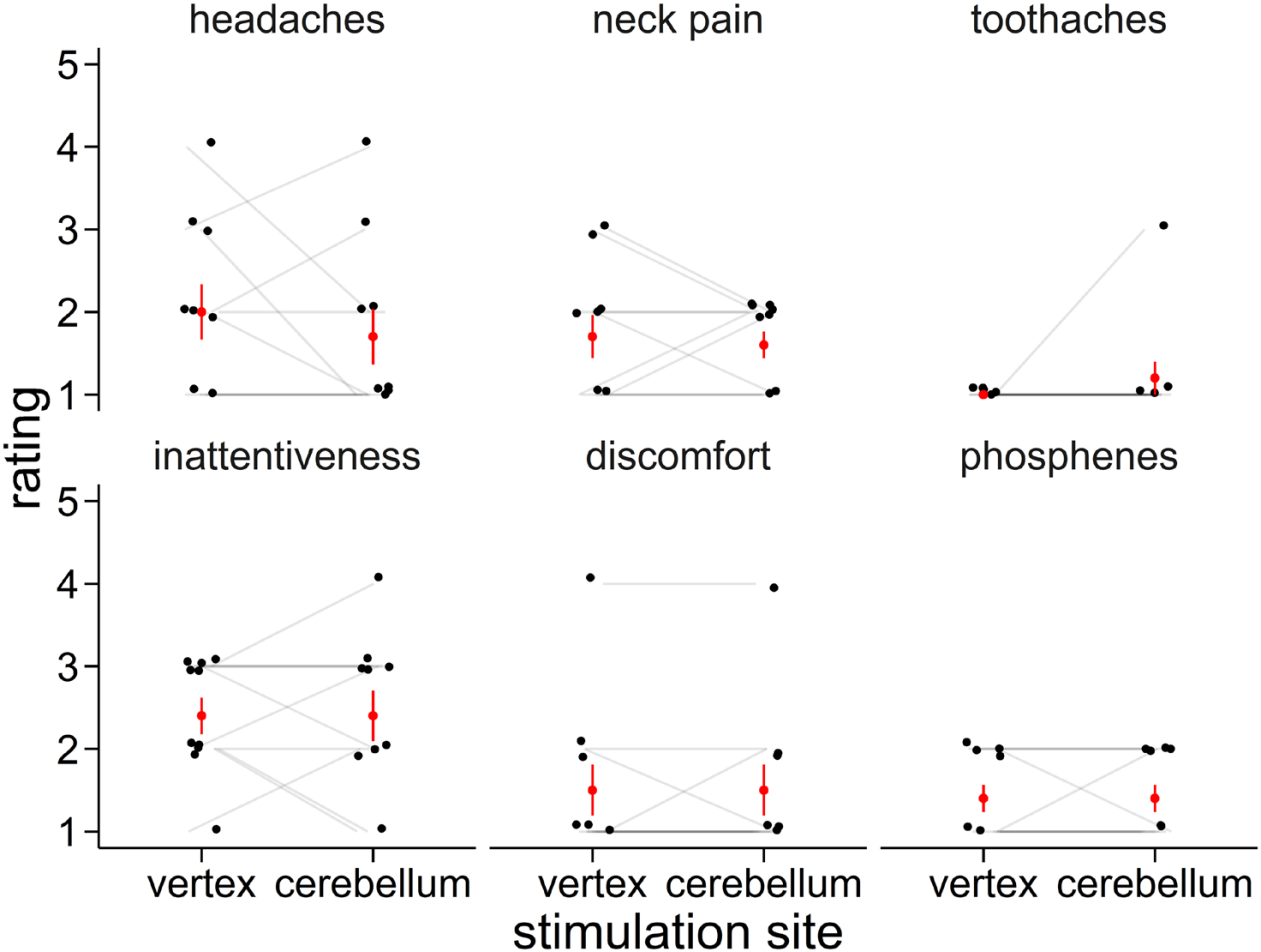
Ratings of side effects in the post‐experimental questionnaire, as reported in Huvermann et al. (2025). Means and standard errors are shown in red, individual ratings are shown in black.

TMS was applied at 120% of motor threshold using a Magstim Double Cone Coil and a Magstim BiStim^2^ unit (Magstim Co., Whitland, United Kingdom). A fast‐paced task flow was enabled by alternating stimulation between two BiStim units. Stimulation was applied either to the left lateral cerebellum (1 cm below and 3 cm to the left of the inion; confer Hardwick et al. 2014; Théoret et al. 2001; Torriero et al. 2004) or position vertex as a control site (at electrode position Cz, Jung et al. 2016; Pizem et al. 2022). Stimulation of the left cerebellar hemisphere is consistent with its implication in processing visual–spatial information (Stoodley and Schmahmann 2009) and stronger activations of the left hemisphere in a previous fMRI study using a similar feedback learning task (Peterburs et al. 2018). Following spontaneous reports of side effects in the initial testing sessions, a post‐experimental questionnaire was introduced in which participants were asked to rate symptoms associated with TMS [see Huvermann et al. (2025) for more details]. No significant differences between vertex and cerebellar stimulation were observed regarding headaches, neck pain, toothaches, inattentiveness, discomfort, phosphenes ratings, or free field responses for other symptoms (all *p* ≥ 0.343, see Figure 3).

### 2.3 EEG Recording and Preprocessing

Data were recorded at 1000 Hz from 30 passive Ag/AgCl multitrode electrodes positioned in the 10–20 system (Chatrian et al. 1985), using BrainAmp MR amplifier and BrainVision Recorder 1.21 (Brain Products GmbH, Gilching, Germany). Impedances were kept below 5 kΩ.

For preprocessing, the ARTIST algorithm by Wu et al. (2018) based on EEGLAB (v2022.1; Delorme and Makeig 2004) was used. This algorithm decreases artifacts in the EEG signal caused by TMS pulses [see Huvermann et al. (2025) for a detailed description of preprocessing procedures].

Using Brainvision Analyzer 2 software (version 2.2, Brain Products GmbH, Gilching, Germany), data were segmented around responses, starting 200 ms before and ending 500 ms after the response. Next, a baseline correction was performed using the time window from 200 to 100 ms before response onset. Data were then exported for further processing in MATLAB. Although data were analyzed on a single‐trial basis, we additionally averaged the data according to conditions (stimulation site, TMS timing, response type) to extract peak latencies of the ERP components of interest (described below). Only trials for stimuli with learnable contingencies (i.e., 80–20) were included.

Peak detection was performed on the averaged data and separately for each condition for the ERN and Pe using MATLAB. The time windows and electrode sites that had been pre‐registered based on previous related studies (Peterburs et al. 2012, 2015; Berlijn et al. 2024a; Tunc et al. 2019) were used. For the ERN, peak detection was performed at FCz in the time window starting at response onset and ending 100 ms thereafter. For the Pe, we used the maximal positive peak within the time window between 200 and 400 ms at Pz. For the single‐trial data, the mean amplitude in a time window around the respective latency determined by the peak detection on the averaged data for each condition was extracted (20 ms for ERN; 40 ms for Pe, Albrecht and Bellebaum 2023; Meadows et al. 2016).

### 2.4 Statistical Data Analysis

Data were analyzed in R (version 4.2.3, R Core Team 2023) using RStudio (version 2023.3.0.386, Posit Team 2023). Analyses of accuracy and choice switching (i.e., choosing a different response than before following e.g., positive/negative feedback) are reported in Huvermann et al. (2025). As data were not clearly separable into pre‐ and post‐learning for a majority of participants, we opted for a single trial‐based analysis approach using linear mixed effects (LME) models including the trial‐by‐trial factor trial number, thus capturing the course of error‐correct differentiation across the experiment. This also overcame a common concern of unequal numbers of trials for errors and correct responses as well as (too) few error trials per condition (Olvet and Hajcak 2009; Pontifex et al. 2010; Larson et al. 2010) due to learning throughout the task. Exclusion of participants with too few error trials would systematically exclude good learners (Clayson et al. 2025) and including an equal number of trials in the analysis does not salvage the concern (Fischer et al. 2017). Multilevel approaches using single‐trial data; however, overcome these limitations by taking into account different numbers of data points per factor level and being relatively robust to large numbers of missing data points (Clayson et al. 2025; Bolker 2015; Krueger and Tian 2004).

The packages lme4 (version 1.1‐32, Bates et al. 2015) and lmertest (version 3.1–3, Kuznetsova et al. 2017) were used for LME modeling. We used restricted maximum likelihood with *p*‐values computed using Satterthwaite approximation to evaluate significance, following Luke (Luke 2017). Participants with a Cook's distance (Cook 1977) above 4/(*n*‐*p*‐1) were identified as outliers (using the influence.ME package, version 0.9‐9, Nieuwenhuis et al. 2012). We strived for a maximal random effects structure but in case of singular fit gradually reduced random effects starting with main effects and then lower‐grade interactions until fit was ensured. Significant interactions were followed up using simple slope analyses (interactions package, version 1.1.5, Long 2019). *p*‐values were Bonferroni‐corrected according to the number of simple slopes.

LME analyses were conducted with the categorical fixed effects response type (−0.5: error, 0.5: correct), stimulation site (−0.5: vertex, 0.5: cerebellum), TMS timing (−0.5: post‐stimulus, 0.5: pre‐feedback), and the continuous factor trial number, which was scaled via the built‐in *scale* function. We also included all interactions of these factors as fixed effects. No participants were identified as outliers based on Cook's distance. The model equation for both ERN and Pe was as follows:

$$\begin{array}{c} ERN/Pe∼1+\text{response type}*\mathrm{TMS}\;\text{condition}*\mathrm{TMS}\;\text{timing}*\text{trial number}\\ +\left(1+\text{response type}:\mathrm{TMS}\;\text{condition}:\mathrm{TMS}\;\text{timing}:\text{trial}\;\text{number}\;|\;\text{subject}\right) \end{array}$$

Note that we also performed a complementary analysis using action value modeling, as commonly conducted for analyses involving prediction error modeling in reinforcement learning contexts (see e.g., McDougle et al. 2019; Ichikawa et al. 2010). Analyses involved a new measure, *Q*_diff_, which reflects the relative subjective action value (action value of the unchosen choice subtracted from the action value of the chosen option). It should thus offer a better measure for subjective error processing than the objective response type (see Supplementary Analysis S2 for further details), especially because, due to the probabilistic nature of our task, errors and correct responses are not as clearly defined as in response‐conflict tasks. Relative action values have been shown to be more reliable than absolute action values (Katahira et al. 2017), although the procedure of action value/prediction error estimation in general has been shown to be highly correlated to subjective measures (Ichikawa et al. 2010).

## 3 Results

### 3.1 Accuracy

While a main effect of block, that is, a general learning effect, was found (*F*(3.18, 73.05) = 6.21, *p* < 0.001), no differences between cerebellar and vertex TMS emerged, *p* ≥ 0.461. These results indicate that error rates decreased over the course of the task and were not affected by cerebellar TMS. On average, 9.6 errors per block, stimulation site, and participant were committed (SD = 4.4 errors). The full results concerning accuracy are reported in Huvermann et al. (2025).

### 3.2 ERN—Effects of Response Type (Error/Correct)

**FIGURE 4:**
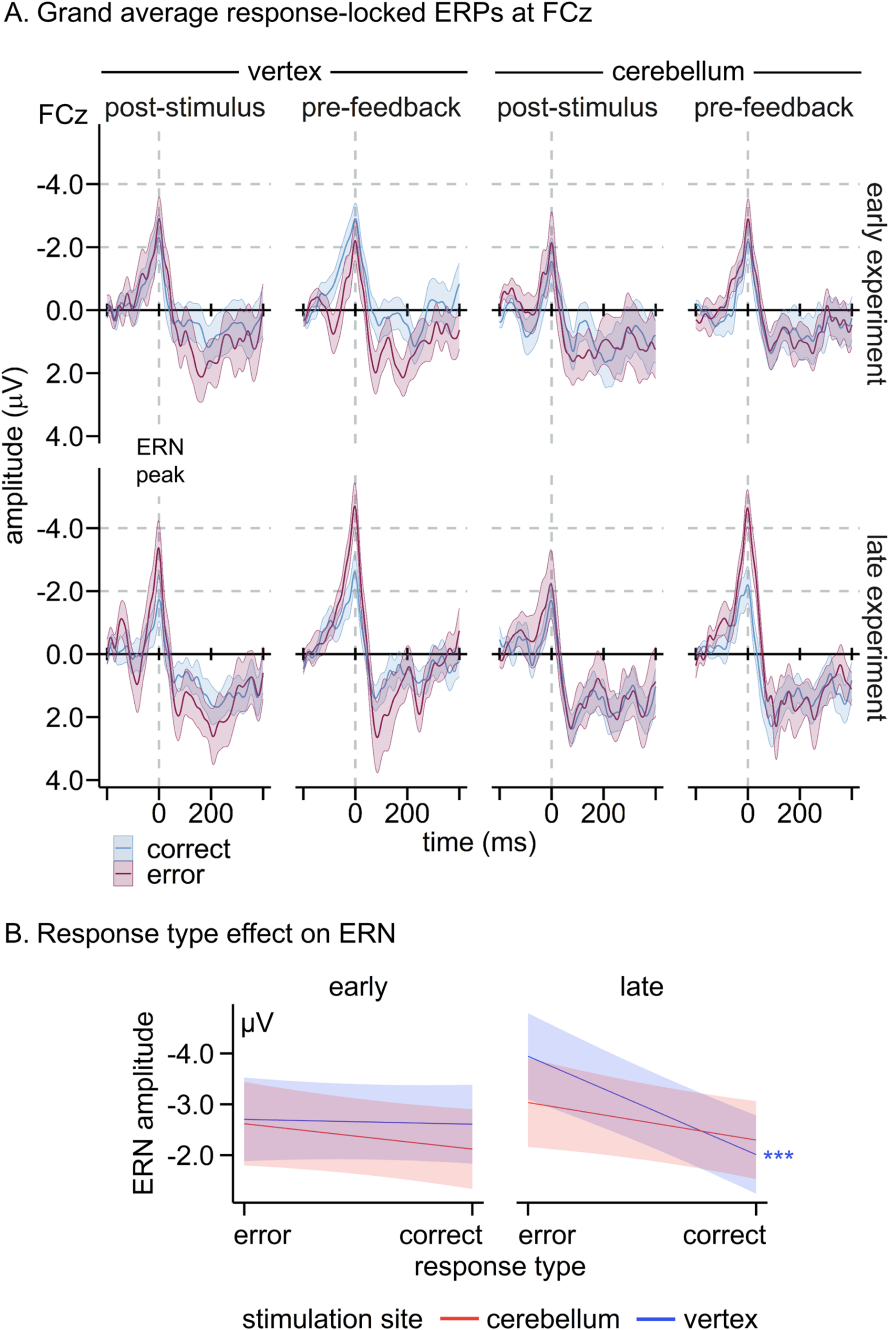
(A) Grand‐average ERPs at FCz locked to individual ERN latencies per condition (response type × stimulation site × TMS timing): early and late in the task according to response type (correct, error), stimulation site (cerebellum, vertex), and TMS timing (post‐stimulus, pre‐feedback). Blue lines denote correct responses, red lines errors. Colored bands display standard errors. See Figure S2 for a response‐locked grand‐average ERP. (B) Slope estimates for ERN amplitude predicted by response type and modulated by stimulation site and trial number (early, late experiment). Red lines denote cerebellar stimulation and blue lines vertex stimulation. Colored bands indicate 95% confidence intervals. ***p < 0.001. *n*_error_ = 2702, *n*_correct_ = 4777.

Grand averages for the ERPs at FCz time‐locked to individual ERN latencies for correct responses and errors (i.e., response type) according to stimulation site, TMS timing, and trial number (early, late experiment) are provided in Figure 4A.

The ERN was more negative for errors compared to correct responses (*β* = 0.81, SE = 0.16, *t*(7451.23) = 4.94, *p* < 0.001). This effect was further modulated by trial number (*β* = 0.51, SE = 0.16, *t*(7394.78) = 3.22, *p* = 0.001). While response types did not differ in ERN amplitude early on (*β* = 0.29, SE = 0.22, *t* = 1.30, *p* = 0.386), errors as compared to correct responses were associated with increased negativity late in the task (*β* = 1.35, SE = 0.24, *t* = 5.75, *p* < 0.001).

Importantly, this interaction was further modulated by stimulation site (*β* = −0.80, SE = 0.32, *t*(7431.03) = 2.53, *p* = 0.012; see Figure 4B). Follow‐up simple‐slope analyses showed that for both cerebellar and vertex TMS, response types were not distinguished in the ERN early in the task (both *p* ≥ 0.453). However, late in the task, the ERN was more pronounced for errors than correct responses for vertex TMS (*β* = 1.94, SE = 0.32, *t* = 6.01, *p* < 0.001) but not for cerebellar TMS (*β* = 0.75, SE = 0.34, *t* = 2.22, *p* = 0.106).

Additionally, a trend‐level interaction between response type, stimulation site, and TMS timing emerged (*β* = 1.22, SE = 0.65, *t*(7448.34) = 1.88, *p* = 0.060; see Figure S1 for the slope plots). Descriptively, response types were distinguished in the ERN for vertex TMS and pre‐feedback cerebellar TMS but not when stimulating the cerebellum post‐stimulus.

Complete inferential statistics are provided in Table S1. Effects that include the TMS timing factor independent of stimulation site are reported in Supplementary Analysis S3.

### 3.3 Pe—Effects of Response Type (Error/Correct)

**FIGURE 5:**
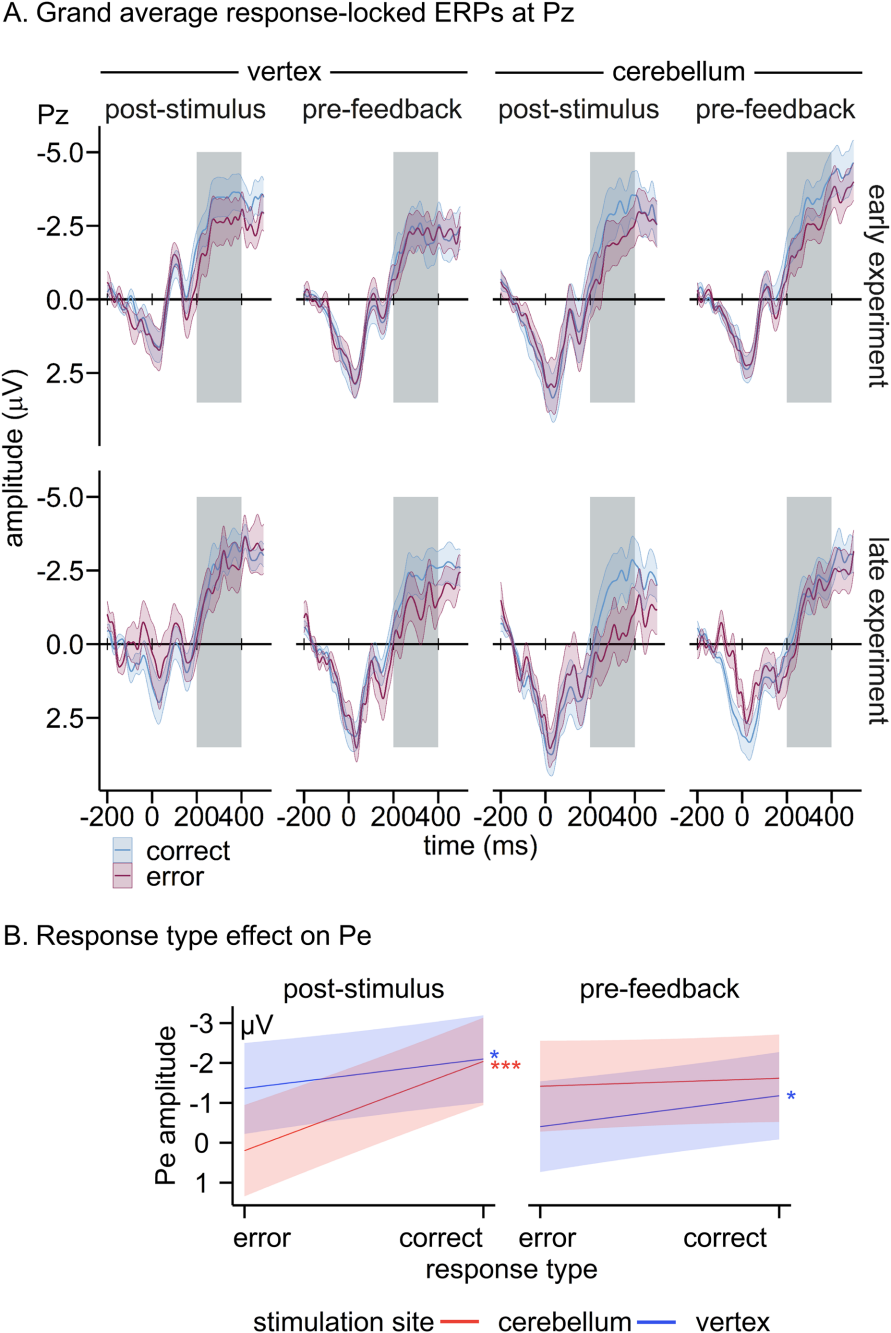
(A) Grand‐average response‐locked ERPs early and late in the task at Pz according to response type (correct, error), stimulation site (cerebellum, vertex) and TMS timing (post‐stimulus, pre‐feedback). Blue lines denote correct responses, red lines errors. Colored bands display standard errors. (B) Slope estimates for Pe amplitude predicted by response type and modulated by stimulation site and TMS timing. Red lines denote cerebellar stimulation and blue lines vertex stimulation. Colored bands indicate 95% confidence intervals. *p < 0.05, ***p < 0.001. *n*_error_ = 2769, *n*_correct_ = 5122.

Grand averages for the response‐locked ERPs at Pz for correct responses and errors (i.e., response type) according to stimulation site, TMS timing, and trial number (early, late experiment) are provided in Figure 5A.

The Pe was more pronounced for errors compared to correct responses (*β* = −0.99, SE = 0.15, *t*(7848.81) = 6.68, *p* < 0.001), and late compared to early in the experiment (*β* = 0.24, SE = 0.07, *t*(7734.12) = 3.37, *p* = 0.001).

Importantly, the effect of response type was modulated by stimulation site and TMS timing (*β* = 2.07, SE = 0.58, *t*(7855.11) = 3.55, *p* < 0.001; see Figure 5B). Post hoc simple slope analyses showed that the Pe differentiated errors and correct responses for vertex TMS applied both pre‐feedback (*β* = −0.76, SE = 0.29, *t* = 2.63, *p* = 0.034) and post‐stimulus (*β* = −0.74, SE = 0.29, *t* = 2.54, *p* = 0.044). For cerebellar TMS, Pe amplitudes did not differ between errors and correct responses when TMS was applied pre‐feedback (*β* = 0.20, SE = 0.29, *t* = 0.70, *p* > 0.999). However, a strong response type effect emerged for cerebellar TMS applied post‐stimulus (*β* = −2.25, SE = 0.30, *t* = 7.58, *p* < 0.001), with more positive amplitudes for errors compared to correct responses. To check whether this response type differentiation in the Pe for post‐stimulus TMS was truly stronger for cerebellar compared to vertex TMS, we checked the interaction effect (stimulation site × response type) for post‐stimulus TMS trials via simple slope analysis, which proved to be significant (*β* = −1.51, SE = 0.41, *t*(7835.18) = 3.64, *p* < 0.001). Notably, the interaction effect did not reach significance for pre‐feedback TMS trials (*β* = 0.55, SE = 0.41, *t*(7838.69) = 1.34, *p* = 0.182), indicating that the differences within post‐stimulus TMS were more decisive for the triple interaction.

Complete inferential statistics can be found in Table S2. Effects that include the TMS timing factor independent of stimulation site are reported in Analysis S3.

### 3.4 Control Analysis—Predictability of Pe by ERN

In an additional analysis we explored whether the effects of spTMS on ERN and Pe were separate effects or whether spTMS only had an effect on ERN which in turn influenced Pe amplitude. The amplitudes of ERN and Pe correlated significantly with each other (*r* = −0.04, *t*(7477) = 3.35, *p* < 0.001), although the correlation strength was very low (Cohen 1988; Evans 1996; Gignac and Szodorai 2016; Funder and Ozer 2019). To check whether the pattern in the Pe is explainable by ERN amplitudes without considering TMS effects, we fitted two additional models: one with the factors response type, trial number, and ERN amplitude (thus disregarding effects of the TMS), and one with the factors response type, trial number, stimulation site, TMS timing, and ERN (thus including both the effects of TMS and ERN). Both models included all interaction terms in the fixed effects. The model including the TMS effects provided a better fit (*χ* ^2^(16) = 116.7, *p* < 0.001) and the triple interaction between response type, stimulation site, and TMS timing remained significant even when ERN was included as an additional factor (*β* = 2.64, SE = 0.65, *t*(7429.14) = 4.05, *p* < 0.001). To examine whether, conversely, the ERN amplitude adds information to the analysis of the Pe, we compared the original model to the model with the ERN as an additional factor. The model fit improved when adding the ERN (*χ* ^2^(16) = 72.60, *p* > 0.001), indicating that the ERN amplitude does explain variance in the Pe amplitude that cannot be explained solely by the other factors. We did not perform the same analysis with ERN amplitude as dependent and Pe as an independent variable as the Pe occurs after the ERN, preventing effects of the Pe onto the ERN (at least within the same trial).

While we used the objective correctness of the responses as a predictor in these analyses (i.e., response type), subjective perception of which action is better/worse might have differed from this, especially considering that responses were associated with outcomes over time in the experiment, that not all participants learned the contingencies and that errors and correct responses were not as clearly defined as in response‐conflict tasks due to the probabilistic nature of action‐outcome associations. We therefore conducted an additional analysis using a measure that reflects the subjective, relative, instead of objective valuation of the chosen option. We computed the *Q*_diff_, that is, the modeled subjective value of the unchosen option subtracted from the value of the chosen option (see Supplementary Analysis S2). This measure thus reflects to what degree the chosen option was perceived as the better/worse option, thereby reflecting intra‐ and interindividual differences in learning and action‐outcome representation (Katahira et al. 2017). Importantly, this analysis yielded a comparable result pattern (see Supplementary Analysis S2).

## 4 Discussion

In the present study, healthy young adults learnt stimulus–response‐feedback associations while single‐pulse TMS (spTMS) was applied to the cerebellum or a control site (vertex) either post‐stimulus (i.e., pre‐response) or pre‐feedback. Response‐related ERP components (ERN and Pe) were analyzed to investigate whether cerebellar output was necessary for error processing in the forebrain during reinforcement learning. Given that feedback processing during reinforcement learning was compromised in cerebellar dysfunction (Huvermann et al. 2025), we expected aberrant error processing for cerebellar TMS. Results in the current study indicate that this is likely the case: Error‐correct differentiation in the ERN was blunted by cerebellar TMS, while being intact for vertex TMS. Error‐correct differentiation in the Pe, on the other hand, was unexpectedly enhanced for post‐stimulus cerebellar TMS.

Consistent with patterns observed in patients with cerebellar damage/dysfunction in a response conflict task (i.e., reduced error‐correct differentiation in the ERN, Peterburs et al. 2012, 2015; Berlijn et al. 2024a), we found reduced error‐correct differentiation in the ERN under cerebellar spTMS. However, the overall result pattern with *unaffected* reinforcement learning (Rustemeier et al. 2016; Thoma et al. 2008), *reduced* error‐correct differentiation in the ERN, and *increased* error‐correct differentiation in the Pe (Peterburs et al. 2012) resembled results observed in patients with cerebellar stroke. The consistency in results between reinforcement learning and response conflict tasks suggests that the cerebellum is involved in error processing in both task contexts in a similar way, in line with its proposed function in performance monitoring (Peterburs and Desmond 2016). Of note, long‐term compensation and/or functional reorganization in stroke recovery have been proposed to support preserved task performance for these patients in a response conflict task (Peterburs et al. 2012). Such effects were previously not observed in patients with progressive cerebellar degeneration who showed an *altered* ERN, *increased* error rates, but *unchanged* Pe in a response conflict task (Peterburs et al. 2015). For the present study, we had expected that cerebellar spTMS disrupts cerebral processing instantaneously (Romero et al. 2019). Long‐term compensation should therefore not be relevant. Instead, increased error‐correct differentiation in the Pe in the presence of reduced differentiation in the ERN was observed instantaneously, giving rise to questions on the underlying mechanisms.

First, it is debatable whether the observed pattern truly represents a compensatory mechanism, or whether the increased differentiation in the Pe could also be the result of hypermetria. This might be the case in terms of a mismatch in salience which is one parameter that correlates with Pe amplitude (Overbeek et al. 2005). Perceived error salience as measured in the Pe might thus be larger than would be appropriate under cerebellar compared to control spTMS. Dysmetria is a common deficit observed in cerebellar disorders (Manto 2009) and has also been suggested as a deficit in cognitive processes (Schmahmann 1998). Future studies could test this by using different error severities/saliencies in their study. An interpretation in terms of hypermetria would indicate that TMS affected ERN and Pe separately from each other. While this might be the case, an indirect effect of TMS on the Pe via the ERN is also conceivable. An additional control analysis indicated that effects within the Pe amplitude are at least partially explainable by ERN amplitude. An indirect effect of TMS on Pe via ERN would be more consistent with an interpretation of the pattern in the Pe in terms of compensation. However, indirect effects via propagation of the TMS stimulus to further brain areas within the same network, as shown for repetitive TMS (Hussain and Freedberg 2025), could provide a further possibility.

While the ERN is generated mostly by the ACC (Debener et al. 2005; Ridderinkhof et al. 2004; Hester et al. 2005; van Veen and Carter 2002; van Boxtel et al. 2005, but also see Herrmann et al. 2004), neural generators for the Pe are less clear and appear not to be limited to the ACC (Overbeek et al. 2005; Hester et al. 2005). This wider network might have allowed the Pe to be less or differently affected by cerebellar spTMS effects, although more conscious error processing as reflected in the Pe may potentially be more effortful and slower. This unexpectedly increased error coding in the Pe might have compensated for deficits in the ERN, allowing unimpeded behavioral performance. Intact behavioral performance was previously not expected due to the instantaneous disruptive effect of spTMS, which in theory does not allow for long‐term compensation as seen in stroke patients (Peterburs et al. 2012). Conversely, differences in properties of the underlying learning mechanism—potentially caused by the deficits in feedback processing/FRN—might have also resulted in differences in error processing later in the task, resulting in decreased use of systems underlying the ERN and increased reliance on systems underlying the Pe, eventually leading to more Pe‐driven error processing. However, these differences in error processing might not always correspond to intact behavioral performance. Relying on later vs. earlier error processing (i.e., on the Pe instead of the ERN) could be unfavorable in everyday tasks that require swift processing, for example, fast‐paced sequences of responses like in sports or music. It is also possible that this potentially compensatory process is not available in all learning contexts, for example, in more complex tasks. Notably, despite overall preserved learning performance, we did find decreased behavioral flexibility (choice switching; see Huvermann et al. 2025), in line with previous findings (Thoma et al. 2008), which might be related to deficits in the ERN.

Concerning the type of cerebellar output essential for ERN but not Pe, our results do not offer a clear answer. In the present dataset, feedback processing was already shown to be impaired (Huvermann et al. 2025). This might have led to impaired adjustments of prediction, resulting in deficits in error processing at the subsequent response stage. However, the effect of cerebellar TMS on ERN and Pe only occurred for post‐stimulus TMS (trend‐level for ERN), which fits better with a perturbation of information processing directly at the response stage. This will likely include predictive processes, as the ERN relies on this rapid matching of representations of the desired and the actual response based on internal information (i.e., an efference copy). The interaction between response type and stimulation in ERN was found only late during the experiment, which also supports predictive processes, as these predictions can only form throughout the learning process. Previous studies in healthy adults could show that error processing in ERN is stronger after learning, while before learning, feedback processing is more dominant (Eppinger et al. 2008; Bellebaum and Colosio 2014; Pietschmann et al. 2008). Perturbed predictive processes would also be consistent with the finding that stimulation timing did not appear to significantly affect feedback processing (Huvermann et al. 2025), as the predictive information is required for updating of predictions at the feedback stage. This mechanism might have affected feedback processing similarly to pre‐feedback TMS. ERN and the FRN (Miltner et al. 1997) are thought to share the ACC as a neural generator (ERN: Dehaene et al. 1994; Miltner et al. 2003; Iannaccone et al. 2015, FRN: Foti et al. 2015; Hauser et al. 2014; Nieuwenhuis et al. 2004), thus potentially being affected in a similar way. Considering the increased error‐correct differentiation in the Pe as a compensatory process, two explanations are possible: The Pe, reflecting more conscious error processing (Wessel 2012; Hester et al. 2005), might either not rely as strongly on cerebellar information, or might also simply be outside the time window of the disruptive effect of the TMS pulse.

Of note, the Pe in our study is not as pronounced as the positive peaks typically found in response conflict paradigms. However, a distinction between errors and correct responses is visible, and a posterior positivity could also be shown in topographical plots of the difference signal for cerebellar post‐stimulus TMS (Figure S3). In two previous studies which examined the Pe in feedback learning paradigms, the Pe peak also seemed to be less prominent in the grand averages (Unger et al. 2012; Zhuang et al. 2021), which might be a characteristic of the Pe in reinforcement learning tasks. This might be due to errors being more ambiguous in feedback learning tasks. However, the Pe is oftentimes not analyzed in feedback learning tasks (Eppinger et al. 2008; Bellebaum and Colosio 2014; Pietschmann et al. 2008; Herbert et al. 2011).

Finally, subjective perception of action values might differ from the objective classification as error/correct. An additional analysis based on action values (*Q*_diff_; Katahira et al. 2017) yielded result patterns consistent with the original results for both ERN and Pe, with reduced *Q*_diff_ differentiation in ERN and increased *Q*_diff_ differentiation in Pe for cerebellar TMS. This demonstrates that the original findings extend to subjective perception of action value, which might be an interesting measure for future studies.

## 5 Limitations

We used an active control site (vertex TMS) instead of sham TMS. While vertex is a common control site in cognitive tasks (e.g., Cao et al. 2021; Ciricugno et al. 2020; Kalbe et al. 2010), at least one study (Jung et al. 2016) showed that vertex TMS reduced activity in the ACC, the likely generator of the ERN (Dehaene et al. 1994; Debener et al. 2005; Ridderinkhof et al. 2004). While we used inverted stimulation which showed considerably less and non‐significant ACC deactivation (Jung et al. 2016), and did not find abnormal ERN patterns during vertex stimulation, we cannot rule out that vertex stimulation affected processing. Unlike Jung et al. (2016), we used a more deeply stimulating double cone coil instead of a figure‐of‐eight coil. Feedback‐related ERP components with neural generators within the ACC seemed to be affected by vertex TMS (Huvermann et al. 2025). Unfortunately, there currently seems to be no well‐tested, better suited site for control stimulation. Sham TMS does not seem ideal as it provides a very different experience regarding vibrations, coil clicks, and magnetic field build (Duecker and Sack 2015). Even though we assessed potential side effects of the TMS stimulation and found no significant differences between vertex and cerebellar stimulation (see Figure 3), we cannot exclude that other differences in the experience of stimulation between the two sites that were not captured, such as stimulation of the neck muscles, emerged and contributed to the findings described above. Future studies may want to include several control sites in between‐subject designs.

Moreover, we only stimulated the left cerebellum. Given that a learning task was used, it was not feasible to repeat the task several times to incorporate other stimulation sites, as repetition effects would have predominated. Future studies should investigate the effect of spTMS on other cerebellar regions in feedback learning using between‐subjects designs.

Last, stimulation was applied either 100 ms post‐stimulus or 100 ms pre‐feedback. There is currently no established time window of cerebellar‐brain inhibition in the cognitive domain as available for the motor domain (Ugawa et al. 1991). Given that stimulation was applied 100 ms post‐stimulus, it usually occurred several hundred milliseconds before the response. Berlijn et al. (2024a) varied stimulation timing around the ERN peak in a Go/NoGo flanker task and found that stimulation at or closely after the calculated peak latency, but not shortly before, decreased error‐correct differentiation, showcasing the time sensitivity of cerebello‐cerebral communication in cognition. This might depend on the task at hand, as in the current study, stimulation before responses also led to altered error processing. Future studies need to explore these temporal dynamics in more detail, for example, by implementing continuous manipulation of stimulation timings.

## 6 Conclusions

The present findings show that cerebellar TMS alters cerebral error processing in reinforcement learning. Error processing was decreased by cerebellar TMS in the ERN and increased in the Pe. This pattern closely resembles altered error processing in cerebellar stroke patients as shown in a previous study in a response conflict task. It remains unclear whether the increased Pe in concert with preserved behavioral performance reflects a compensatory process. Processing was affected more strongly by stimulation closer in time to response execution (i.e., post‐stimulus/pre‐response). Taken together, the present study adds to a growing body of evidence showing that the cerebellum plays an important role in error processing and performance monitoring in general, whereby it directly contributes to reinforcement learning and adaptive control of behavior.

## Author Contributions

**Dana M. Huvermann:** conceptualization, methodology, software, formal analysis, investigation, data curation, writing – original draft, visualization, project administration. **Adam M. Berlijn:** conceptualization, methodology, software, validation, formal analysis, investigation, data curation, writing – review and editing, project administration. **Stefan J. Groiss:** conceptualization, methodology, resources, writing – review and editing, supervision, project administration. **Manfred Mittelstaedt:** software, resources, writing – review and editing. **Alfons Schnitzler:** resources, writing – review and editing. **Christian Bellebaum:** conceptualization, methodology, validation, resources, writing – review and editing, supervision. **Martina Minnerop:** conceptualization, writing – review and editing, supervision, funding acquisition. **Dagmar Timmann:** conceptualization, resources, writing – review and editing, supervision, funding acquisition. **Jutta Peterburs:** conceptualization, methodology, validation, resources, writing – review and editing, supervision, funding acquisition.

## Ethics Statement

The study was conducted in accordance with the ethical principles for medical research involving human subjects outlined in the Declaration of Helsinki and approved by the Ethics Committees at the Faculty of Medicine of Heinrich‐Heine‐University Düsseldorf (2018‐240_1) and the University Hospital Essen (18‐8477‐BO).

## Conflicts of Interest

The authors declare no conflicts of interest.

## Supporting information


**Data S1:** psyp70178‐sup‐0001‐Supinfo.docx.

## Data Availability

The experimental protocol was defined prior to experiments and preregistered to the open science framework (OSF, see https://doi.org/10.17605/OSF.IO/A24RG for the TMS study and https://doi.org/10.17605/OSF.IO/RD3XB for the patient study). Raw data used in the analysis is openly available at https://osf.io/9n7yp.

## References

[psyp70178-bib-0001] Albrecht, C. , and C. Bellebaum . 2023. “Slip or Fallacy? Effects of Error Severity on Own and Observed Pitch Error Processing in Pianists.” Cognitive, Affective, & Behavioral Neuroscience 23, no. 4: 1076–1094. 10.3758/s13415-023-01097-1 PMC10400674 37198385

[psyp70178-bib-0002] Bates, D. , M. Mächler , B. Bolker , and S. Walker . 2015. “Fitting Linear Mixed‐Effects Models Using lme4.” Journal of Statistical Software 67, no. 1: 1–48.

[psyp70178-bib-0003] Bellebaum, C. , and M. Colosio . 2014. “From Feedback‐ to Response‐Based Performance Monitoring in Active and Observational Learning.” Journal of Cognitive Neuroscience 26, no. 9: 2111–2127. 24666168 10.1162/jocn_a_00612

[psyp70178-bib-0004] Berlijn, A. M. , D. M. Huvermann , E. Bechler , et al. 2025. “Impaired Reinforcement Learning and Coding of Prediction Errors in Patients With Cerebellar Degeneration ‐ a Study With EEG and Voxel‐Based Morphometry.” Cognitive, Affective, & Behavioral Neuroscience 25: 1126–1146. 10.3758/s13415-025-01303-2 PMC12356735 40437311

[psyp70178-bib-0005] Berlijn, A. M. , D. M. Huvermann , S. J. Groiss , et al. 2024a. “The Effect of Cerebellar TMS on Error Processing: A Combined Single‐Pulse TMS and ERP Study.” Imaging Neuroscience 2: 1–19.

[psyp70178-bib-0006] Berlijn, A. M. , D. M. Huvermann , S. Schneider , et al. 2024b. “The Role of the Human Cerebellum for Learning From and Processing of External Feedback in Non‐Motor Learning: A Systematic Review.” Cerebellum 23: 1532–1551. 38379034 10.1007/s12311-024-01669-y PMC11269477

[psyp70178-bib-0007] Bolker, B. M. 2015. “Linear and Generalized Linear Mixed Models.” Ecological Statistics: Contemporary Theory and Application 2015: 309–333.

[psyp70178-bib-0008] Bostan, A. C. , and P. L. Strick . 2018. “The Basal Ganglia and the Cerebellum: Nodes in an Integrated Network.” Nature Reviews Neuroscience 19, no. 6: 338–350. 29643480 10.1038/s41583-018-0002-7 PMC6503669

[psyp70178-bib-0009] Cao, D. , Y. Li , and Y. Tang . 2021. “Functional Specificity of the Left Ventrolateral Prefrontal Cortex in Positive Reappraisal: A Single‐Pulse Transcranial Magnetic Stimulation Study.” Cognitive, Affective, & Behavioral Neuroscience 21, no. 4: 793–804. 10.3758/s13415-021-00881-1 33751480

[psyp70178-bib-0010] Chatrian, G. E. , E. Lettich , and P. L. Nelson . 1985. “Ten Percent Electrode System for Topographic Studies of Spontaneous and Evoked EEG Activities.” American Journal of EEG Technology 25, no. 2: 83–92.

[psyp70178-bib-0011] Chen, C. , T. Takahashi , S. Nakagawa , T. Inoue , and I. Kusumi . 2015. “Reinforcement Learning in Depression: A Review of Computational Research.” Neuroscience and Biobehavioral Reviews 55: 247–267. 25979140 10.1016/j.neubiorev.2015.05.005

[psyp70178-bib-0012] Ciricugno, A. , C. Ferrari , M. L. Rusconi , and Z. Cattaneo . 2020. “The Left Posterior Cerebellum Is Involved in Orienting Attention Along the Mental Number Line: An Online‐TMS Study.” Neuropsychologia 143: 107497. 32413432 10.1016/j.neuropsychologia.2020.107497

[psyp70178-bib-0013] Clayson, P. E. , S. A. Baldwin , and M. J. Larson . 2025. “Stability of Performance Monitoring With Prolonged Task Performance: A Study of Error‐Related Negativity and Error Positivity.” Psychophysiology 62, no. 2: e14731. 39655436 10.1111/psyp.14731

[psyp70178-bib-0014] Cohen, J. 1988. Statistical Power Analysis for the Behavioral Sciences. 2nd ed. Routledge. 10.4324/9780203771587.

[psyp70178-bib-0015] Coles, M. G. H. , M. K. Scheffers , and C. B. Holroyd . 2001. “Why Is There an ERN/ne on Correct Trials? Response Representations, Stimulus‐Related Components, and the Theory of Error‐Processing.” Biological Psychology 56, no. 3: 173–189. 11399349 10.1016/s0301-0511(01)00076-x

[psyp70178-bib-0016] Cook, R. D. 1977. “Detection of Influential Observation in Linear Regression.” Technometrics 19, no. 1: 15–18.

[psyp70178-bib-0017] Corlett, P. R. , J. A. Mollick , and H. Kober . 2022. “Meta‐Analysis of Human Prediction Error for Incentives, Perception, Cognition, and Action.” Neuropsychopharmacology 47, no. 7: 1339–1349. 35017672 10.1038/s41386-021-01264-3 PMC9117315

[psyp70178-bib-0018] Debener, S. , M. Ullsperger , M. Siegel , K. Fiehler , D. Y. Von Cramon , and A. K. Engel . 2005. “Trial‐by‐Trial Coupling of Concurrent Electroencephalogram and Functional Magnetic Resonance Imaging Identifies the Dynamics of Performance Monitoring.” Journal of Neuroscience 25, no. 50: 11730–11737. 16354931 10.1523/JNEUROSCI.3286-05.2005 PMC6726024

[psyp70178-bib-0019] Dehaene, S. , M. I. Posner , and D. M. Tucker . 1994. “Localization of a Neural System for Error Detection and Compensation.” Psychological Science 5, no. 5: 303–305.

[psyp70178-bib-0020] Delorme, A. , and S. Makeig . 2004. “EEGLAB: An Open‐Source Toolbox for Analysis of Single‐Trial EEG Dynamics.” Journal of Neuroscience Methods 134: 9–21. 15102499 10.1016/j.jneumeth.2003.10.009

[psyp70178-bib-0021] Desmond, J. E. , S. H. A. Chen , and P. B. Shieh . 2005. “Cerebellar Transcranial Magnetic Stimulation Impairs Verbal Working Memory.” Annals of Neurology 58, no. 4: 553–560. 16178033 10.1002/ana.20604

[psyp70178-bib-0022] Du, X. , L. M. Rowland , A. Summerfelt , et al. 2018. “Cerebellar‐Stimulation Evoked Prefrontal Electrical Synchrony Is Modulated by GABA.” Cerebellum 17, no. 5: 550–563. 29766458 10.1007/s12311-018-0945-2 PMC6237666

[psyp70178-bib-0023] Duecker, F. , and A. T. Sack . 2015. “Rethinking the Role of Sham TMS.” Frontiers in Psychology 6: 210. 25767458 10.3389/fpsyg.2015.00210 PMC4341423

[psyp70178-bib-0024] Eppinger, B. , J. Kray , B. Mock , and A. Mecklinger . 2008. “Better or Worse Than Expected? Aging, Learning, and the ERN.” Neuropsychologia 46, no. 2: 521–539. 17936313 10.1016/j.neuropsychologia.2007.09.001

[psyp70178-bib-0025] Evans, J. D. 1996. Straightforward Statistics for the Behavioral Sciences. Thomson Brooks/Cole Publishing Co.

[psyp70178-bib-0026] Falkenstein, M. , J. Hohnsbein , J. Hoormann , and L. Blanke . 1991. “Effects of Crossmodal Divided Attention on Late ERP Components. II. Error Processing in Choice Reaction Tasks.” Electroencephalography and Clinical Neurophysiology 78, no. 6: 447–455. 1712280 10.1016/0013-4694(91)90062-9

[psyp70178-bib-0027] Fernandez, L. , N. C. Rogasch , M. Do , et al. 2020. “Cerebral Cortical Activity Following Non‐Invasive Cerebellar Stimulation—A Systematic Review of Combined TMS and EEG Studies.” Cerebellum 19, no. 2: 309–335. 31907864 10.1007/s12311-019-01093-7

[psyp70178-bib-0028] Fischer, A. G. , T. A. Klein , and M. Ullsperger . 2017. “Comparing the Error‐Related Negativity Across Groups: The Impact of Error‐ and Trial‐Number Differences.” Psychophysiology 54, no. 7: 998–1009. 28369880 10.1111/psyp.12863

[psyp70178-bib-0029] Foti, D. , A. Weinberg , E. M. Bernat , and G. H. Proudfit . 2015. “Anterior Cingulate Activity to Monetary Loss and Basal Ganglia Activity to Monetary Gain Uniquely Contribute to the Feedback Negativity.” Clinical Neurophysiology 126, no. 7: 1338–1347. 25454338 10.1016/j.clinph.2014.08.025 PMC4385748

[psyp70178-bib-0030] Funder, D. C. , and D. J. Ozer . 2019. “Evaluating Effect Size in Psychological Research: Sense and Nonsense.” Advances in Methods and Practices in Psychological Science 2, no. 2: 156–168.

[psyp70178-bib-0031] Gehring, W. J. , B. Goss , M. G. H. Coles , D. E. Meyer , and E. Donchin . 1993. “A Neural System for Error Detection and Compensation.” Psychological Science 4, no. 6: 385–390.

[psyp70178-bib-0032] Gentsch, A. , P. Ullsperger , and M. Ullsperger . 2009. “Dissociable Medial Frontal Negativities From a Common Monitoring System for Self‐ and Externally Caused Failure of Goal Achievement.” NeuroImage 47, no. 4: 2023–2030. 19486945 10.1016/j.neuroimage.2009.05.064

[psyp70178-bib-0033] Gignac, G. E. , and E. T. Szodorai . 2016. “Effect Size Guidelines for Individual Differences Researchers.” Personality and Individual Differences 102: 74–78.

[psyp70178-bib-0034] Glickstein, M. , J. G. May III , and B. E. Mercier . 1985. “Corticopontine Projection in the Macaque: The Distribution of Labelled Cortical Cells After Large Injections of Horseradish Peroxidase in the Pontine Nuclei.” Journal of Comparative Neurology 235, no. 3: 343–359. 3998215 10.1002/cne.902350306

[psyp70178-bib-0035] Gueguen, M. C. , E. M. Schweitzer , and A. B. Konova . 2021. “Computational Theory‐Driven Studies of Reinforcement Learning and Decision‐Making in Addiction: What Have We Learned?” Current Opinion in Behavioral Sciences 38: 40–48. 34423103 10.1016/j.cobeha.2020.08.007 PMC8376201

[psyp70178-bib-0036] Habas, C. 2021. “Functional Connectivity of the Cognitive Cerebellum.” Frontiers in Systems Neuroscience 15: 642225. 33897382 10.3389/fnsys.2021.642225 PMC8060696

[psyp70178-bib-0037] Hardwick, R. M. , E. Lesage , and R. C. Miall . 2014. “Cerebellar Transcranial Magnetic Stimulation: The Role of Coil Geometry and Tissue Depth.” Brain Stimulation 7, no. 5: 643–649. 24924734 10.1016/j.brs.2014.04.009 PMC4180011

[psyp70178-bib-0038] Hauser, T. U. , R. Iannaccone , P. Stämpfli , et al. 2014. “The Feedback‐Related Negativity (FRN) Revisited: New Insights Into the Localization, Meaning and Network Organization.” NeuroImage 84: 159–168. 23973408 10.1016/j.neuroimage.2013.08.028

[psyp70178-bib-0039] Herbert, M. , B. Eppinger , and J. Kray . 2011. “Younger but Not Older Adults Benefit From Salient Feedback During Learning.” Frontiers in Psychology 2: 171. 10.3389/fpsyg.2011.00171. 21886630 PMC3154404

[psyp70178-bib-0040] Herrmann, M. J. , J. Römmler , A. C. Ehlis , A. Heidrich , and A. J. Fallgatter . 2004. “Source Localization (LORETA) of the Error‐Related‐Negativity (ERN/Ne) and Positivity (Pe).” Cognitive Brain Research 20, no. 2: 294–299. 15183400 10.1016/j.cogbrainres.2004.02.013

[psyp70178-bib-0041] Hester, R. , C. Fassbender , and H. Garavan . 2004. “Individual Differences in Error Processing: A Review and Reanalysis of Three Event‐Related fMRI Studies Using the GO/NOGO Task.” Cerebral Cortex 14, no. 9: 986–994. 15115734 10.1093/cercor/bhh059

[psyp70178-bib-0042] Hester, R. , J. J. Foxe , S. Molholm , M. Shpaner , and H. Garavan . 2005. “Neural Mechanisms Involved in Error Processing: A Comparison of Errors Made With and Without Awareness.” NeuroImage 27, no. 3: 602–608. 16024258 10.1016/j.neuroimage.2005.04.035

[psyp70178-bib-0043] Holroyd, C. B. , and M. G. H. Coles . 2002. “The Neural Basis of Human Error Processing: Reinforcement Learning, Dopamine, and the Error‐Related Negativity.” Psychological Review 109, no. 4: 679–709. 12374324 10.1037/0033-295X.109.4.679

[psyp70178-bib-0044] Hussain, S. J. , and M. V. Freedberg . 2025. “Debunking the Myth of Excitatory and Inhibitory Repetitive Transcranial Magnetic Stimulation in Cognitive Neuroscience Research.” Journal of Cognitive Neuroscience 37, no. 5: 1009–1022. 39785679 10.1162/jocn_a_02288

[psyp70178-bib-0045] Huvermann, D. , A. Berlijn , A. Thieme , et al. 2025. “The Cerebellum Contributes to Prediction Error Coding in Reinforcement Learning in Humans.” Journal of Neuroscience 45: e1972242025. 40139806 10.1523/JNEUROSCI.1972-24.2025 PMC12060651

[psyp70178-bib-0046] Iannaccone, R. , T. U. Hauser , P. Staempfli , S. Walitza , D. Brandeis , and S. Brem . 2015. “Conflict Monitoring and Error Processing: New Insights From Simultaneous EEG–fMRI.” NeuroImage 105: 395–407. 25462691 10.1016/j.neuroimage.2014.10.028

[psyp70178-bib-0047] Ichikawa, N. , G. J. Siegle , A. Dombrovski , and H. Ohira . 2010. “Subjective and Model‐Estimated Reward Prediction: Association With the Feedback‐Related Negativity (FRN) and Reward Prediction Error in a Reinforcement Learning Task.” International Journal of Psychophysiology 78, no. 3: 273–283. 20858518 10.1016/j.ijpsycho.2010.09.001 PMC3150511

[psyp70178-bib-0048] Jung, J. , A. Bungert , R. Bowtell , and S. R. Jackson . 2016. “Vertex Stimulation as a Control Site for Transcranial Magnetic Stimulation: A Concurrent TMS/fMRI Study.” Brain Stimulation 9, no. 1: 58–64. 26508284 10.1016/j.brs.2015.09.008 PMC4720218

[psyp70178-bib-0049] Kalbe, E. , M. Schlegel , A. T. Sack , et al. 2010. “Dissociating Cognitive From Affective Theory of Mind: A TMS Study.” Cortex 46, no. 6: 769–780. 19709653 10.1016/j.cortex.2009.07.010

[psyp70178-bib-0050] Katahira, K. , S. Yuki , and K. Okanoya . 2017. “Model‐Based Estimation of Subjective Values Using Choice Tasks With Probabilistic Feedback.” Journal of Mathematical Psychology 79: 29–43.

[psyp70178-bib-0051] Kostadinov, D. , and M. Häusser . 2022. “Reward Signals in the Cerebellum: Origins, Targets, and Functional Implications.” Neuron 110, no. 8: 1290–1303. 35325616 10.1016/j.neuron.2022.02.015

[psyp70178-bib-0052] Krueger, C. , and L. Tian . 2004. “A Comparison of the General Linear Mixed Model and Repeated Measures ANOVA Using a Dataset With Multiple Missing Data Points.” Biological Research for Nursing 6, no. 2: 151–157. 15388912 10.1177/1099800404267682

[psyp70178-bib-0053] Kruithof, E. S. , J. Klaus , and D. J. L. G. Schutter . 2023. “The Human Cerebellum in Reward Anticipation and Outcome Processing: An Activation Likelihood Estimation Meta‐Analysis.” Neuroscience and Biobehavioral Reviews 149: 105171. 37060968 10.1016/j.neubiorev.2023.105171

[psyp70178-bib-0054] Kuznetsova, A. , P. Brockhoff , and R. Christensen . 2017. “lmerTest Package: Tests in Linear Mixed Effects Models.” Journal of Statistical Software 82, no. 13: 1–26.

[psyp70178-bib-0055] Larson, M. J. , S. A. Baldwin , D. A. Good , and J. E. Fair . 2010. “Brief Reports: Temporal Stability of the Error‐Related Negativity (ERN) and Post‐Error Positivity (Pe): The Role of Number of Trials.” Psychophysiology 47, no. 6: 1167–1171. 20477982 10.1111/j.1469-8986.2010.01022.x

[psyp70178-bib-0056] Long, J. A. 2019. “Interactions: Comprehensive, User‐Friendly Toolkit for Probing Interactions [Internet].” https://cran.r‐project.org/package=interactions.

[psyp70178-bib-0057] Luber, B. , and S. H. Lisanby . 2014. “Enhancement of Human Cognitive Performance Using Transcranial Magnetic Stimulation (TMS).” Neuroimage 85: 961–970. 23770409 10.1016/j.neuroimage.2013.06.007 PMC4083569

[psyp70178-bib-0058] Luke, S. G. 2017. “Evaluating Significance in Linear Mixed‐Effects Models in R.” Behavior Research Methods 49, no. 4: 1494–1502. 27620283 10.3758/s13428-016-0809-y

[psyp70178-bib-0059] Manto, M. U. 2009. “Mechanisms of Human Cerebellar Dysmetria: Experimental Evidence and Current Conceptual Bases.” Journal of NeuroEngineering and Rehabilitation 6, no. 1: 10. 19364396 10.1186/1743-0003-6-10 PMC2679756

[psyp70178-bib-0060] McDougle, S. D. , P. A. Butcher , D. E. Parvin , et al. 2019. “Neural Signatures of Prediction Errors in a Decision‐Making Task Are Modulated by Action Execution Failures.” Current Biology 29, no. 10: 1606–1613. 31056386 10.1016/j.cub.2019.04.011 PMC6535105

[psyp70178-bib-0061] Meadows, C. C. , P. A. Gable , K. R. Lohse , and M. W. Miller . 2016. “The Effects of Reward Magnitude on Reward Processing: An Averaged and Single Trial Event‐Related Potential Study.” Biological Psychology 118: 154–160. 27288743 10.1016/j.biopsycho.2016.06.002

[psyp70178-bib-0062] Miltner, W. H. R. , C. H. Braun , and M. G. H. Coles . 1997. “Event‐Related Brain Potentials Following Incorrect Feedback in a Time‐Estimation Task: Evidence for a ‘Generic’ Neural System for Error Detection.” Journal of Cognitive Neuroscience 9, no. 6: 788–798. 23964600 10.1162/jocn.1997.9.6.788

[psyp70178-bib-0063] Miltner, W. H. R. , U. Lemke , T. Weiss , C. Holroyd , M. K. Scheffers , and M. G. H. Coles . 2003. “Implementation of Error‐Processing in the Human Anterior Cingulate Cortex: A Source Analysis of the Magnetic Equivalent of the Error‐Related Negativity.” Biological Psychology 64, no. 1: 157–166. 14602360 10.1016/s0301-0511(03)00107-8

[psyp70178-bib-0064] Nieuwenhuis, R. , M. te Grotenhuis , and B. Pelzer . 2012. “Influence.ME: Tools for Detecting Influential Data in Mixed Effects Models.” R Journal 4, no. 2: 38–47.

[psyp70178-bib-0065] Nieuwenhuis, S. , C. B. Holroyd , N. Mol , and M. G. H. Coles . 2004. “Reinforcement‐Related Brain Potentials From Medial Frontal Cortex: Origins and Functional Significance.” Neuroscience and Biobehavioral Reviews 28, no. 4: 441–448. 15289008 10.1016/j.neubiorev.2004.05.003

[psyp70178-bib-0066] Nieuwenhuis, S. , K. R. Ridderinkhof , J. Blom , G. P. H. Band , and A. Kok . 2001. “Error‐Related Brain Potentials Are Differentially Related to Awareness of Response Errors: Evidence From an Antisaccade Task.” Psychophysiology 38, no. 5: 752–760. 11577898

[psyp70178-bib-0067] Oldfield, R. C. 1971. “The Assessment and Analysis of Handedness: The Edinburgh Inventory.” Neuropsychologia 9, no. 1: 97–113. 5146491 10.1016/0028-3932(71)90067-4

[psyp70178-bib-0068] Olvet, D. M. , and G. Hajcak . 2009. “The Stability of Error‐Related Brain Activity With Increasing Trials.” Psychophysiology 46, no. 5: 957–961. 19558398 10.1111/j.1469-8986.2009.00848.x

[psyp70178-bib-0069] Overbeek, T. J. M. , S. Nieuwenhuis , and K. R. Ridderinkhof . 2005. “Dissociable Components of Error Processing.” Journal of Psychophysiology 19, no. 4: 319–329.

[psyp70178-bib-0070] Peterburs, J. , and J. E. Desmond . 2016. “The Role of the Human Cerebellum in Performance Monitoring.” Current Opinion in Neurobiology 40: 38–44. 27372055 10.1016/j.conb.2016.06.011 PMC5056810

[psyp70178-bib-0071] Peterburs, J. , K. Gajda , B. Koch , et al. 2012. “Cerebellar Lesions Alter Performance Monitoring on the Antisaccade Task—An Event‐Related Potentials Study.” Neuropsychologia 50, no. 3: 379–389. 22227094 10.1016/j.neuropsychologia.2011.12.009

[psyp70178-bib-0072] Peterburs, J. , D. Hofmann , M. P. I. Becker , A. M. Nitsch , W. H. R. Miltner , and T. Straube . 2018. “The Role of the Cerebellum for Feedback Processing and Behavioral Switching in a Reversal‐Learning Task.” Brain and Cognition 125: 142–148. 29990704 10.1016/j.bandc.2018.07.001

[psyp70178-bib-0073] Peterburs, J. , M. Thürling , M. Rustemeier , et al. 2015. “A Cerebellar Role in Performance Monitoring–Evidence From EEG and Voxel‐Based Morphometry in Patients With Cerebellar Degenerative Disease.” Neuropsychologia 68: 139–147. 25592368 10.1016/j.neuropsychologia.2015.01.017

[psyp70178-bib-0074] Pietschmann, M. , K. Simon , T. Endrass , and N. Kathmann . 2008. “Changes of Performance Monitoring With Learning in Older and Younger Adults.” Psychophysiology 45, no. 4: 559–568. 18266802 10.1111/j.1469-8986.2008.00651.x

[psyp70178-bib-0075] Pizem, D. , L. Novakova , M. Gajdos , and I. Rektorova . 2022. “Is the Vertex a Good Control Stimulation Site? Theta Burst Stimulation in Healthy Controls.” Journal of Neural Transmission 129, no. 3: 319–329. 35076779 10.1007/s00702-022-02466-9

[psyp70178-bib-0076] Pontifex, M. B. , M. R. Scudder , M. L. Brown , et al. 2010. “On the Number of Trials Necessary for Stabilization of Error‐Related Brain Activity Across the Life Span.” Psychophysiology 47, no. 4: 767–773. 20230502 10.1111/j.1469-8986.2010.00974.x

[psyp70178-bib-0077] Popa, L. S. , and T. J. Ebner . 2019. “Cerebellum, Predictions and Errors.” Frontiers in Cellular Neuroscience 12: 524. 30697149 10.3389/fncel.2018.00524 PMC6340992

[psyp70178-bib-0078] Posit Team . 2023. RStudio: Integrated Development Environment for R [Internet]. Posit Software, PBC. http://www.posit.co/.

[psyp70178-bib-0079] Potts, G. F. , L. E. Martin , S. M. Kamp , and E. Donchin . 2011. “Neural Response to Action and Reward Prediction Errors: Comparing the Error‐Related Negativity to Behavioral Errors and the Feedback‐Related Negativity to Reward Prediction Violations.” Psychophysiology 48, no. 2: 218–228. 20557487 10.1111/j.1469-8986.2010.01049.x PMC2965315

[psyp70178-bib-0080] R Core Team . 2023. R: A Language and Environment for Statistical Computing [Internet]. R Foundation for Statistical Computation. https://www.R‐project.org/.

[psyp70178-bib-0081] Ramnani, N. 2012. “Frontal Lobe and Posterior Parietal Contributions to the Cortico‐Cerebellar System.” Cerebellum 11, no. 2: 366–383. 21671065 10.1007/s12311-011-0272-3

[psyp70178-bib-0082] Ridderinkhof, K. R. , J. R. Ramautar , and J. G. Wijnen . 2009. “To PE or Not to PE: A P3‐Like ERP Component Reflecting the Processing of Response Errors.” Psychophysiology 46, no. 3: 531–538. 19226310 10.1111/j.1469-8986.2009.00790.x

[psyp70178-bib-0083] Ridderinkhof, K. R. , M. Ullsperger , E. A. Crone , and S. Nieuwenhuis . 2004. “The Role of the Medial Frontal Cortex in Cognitive Control.” Science 306, no. 5695: 443–447. 15486290 10.1126/science.1100301

[psyp70178-bib-0084] Romero, M. C. , M. Davare , M. Armendariz , and P. Janssen . 2019. “Neural Effects of Transcranial Magnetic Stimulation at the Single‐Cell Level.” Nature Communications 10, no. 1: 2642. 10.1038/s41467-019-10638-7 PMC6572776 31201331

[psyp70178-bib-0085] Rustemeier, M. , B. Koch , M. Schwarz , and C. Bellebaum . 2016. “Processing of Positive and Negative Feedback in Patients With Cerebellar Lesions.” Cerebellum 15: 425–438. 26208703 10.1007/s12311-015-0702-8

[psyp70178-bib-0086] Schmahmann, J. D. 1998. “Dysmetria of Thought: Clinical Consequences of Cerebellar Dysfunction on Cognition and Affect.” Trends in Cognitive Sciences 2, no. 9: 362–371. 21227233 10.1016/s1364-6613(98)01218-2

[psyp70178-bib-0087] Schmahmann, J. D. , and D. N. Pandya . 1997. “Anatomic Organization of the Basilar Pontine Projections From Prefrontal Cortices in Rhesus Monkey.” Journal of Neuroscience 17, no. 1: 438–458. 8987769 10.1523/JNEUROSCI.17-01-00438.1997 PMC6793685

[psyp70178-bib-0088] Schutter, D. J. L. G. , and J. van Honk . 2006. “An Electrophysiological Link Between the Cerebellum, Cognition and Emotion: Frontal Theta EEG Activity to Single‐Pulse Cerebellar TMS.” NeuroImage 33, no. 4: 1227–1231. 17023183 10.1016/j.neuroimage.2006.06.055

[psyp70178-bib-0089] Shirota, Y. , and Y. Ugawa . 2024. “Transcranial Magnetic Stimulation.” Current Opinion in Behavioral Sciences 58: 101396.

[psyp70178-bib-0090] Sokolov, A. A. , R. C. Miall , and R. B. Ivry . 2017. “The Cerebellum: Adaptive Prediction for Movement and Cognition.” Trends in Cognitive Sciences 21, no. 5: 313–332. 28385461 10.1016/j.tics.2017.02.005 PMC5477675

[psyp70178-bib-0091] Stoodley, C. J. , and J. D. Schmahmann . 2009. “Functional Topography in the Human Cerebellum: A Meta‐Analysis of Neuroimaging Studies.” NeuroImage 44, no. 2: 489–501. 18835452 10.1016/j.neuroimage.2008.08.039

[psyp70178-bib-0092] Sutton, R. S. , and A. G. Barto . 2018. Reinforcement Learning: An Introduction. MIT Press.

[psyp70178-bib-0093] Tanaka, H. , T. Ishikawa , J. Lee , and S. Kakei . 2020. “The Cerebro‐Cerebellum as a Locus of Forward Model: A Review.” Frontiers in Systems Neuroscience 14: 19. 10.3389/fnsys.2020.00019. 32327978 PMC7160920

[psyp70178-bib-0094] Théoret, H. , J. Haque , and A. Pascual‐Leone . 2001. “Increased Variability of Paced Finger Tapping Accuracy Following Repetitive Magnetic Stimulation of the Cerebellum in Humans.” Neuroscience Letters 306, no. 1: 29–32. 11403950 10.1016/s0304-3940(01)01860-2

[psyp70178-bib-0095] Thoma, P. , C. Bellebaum , B. Koch , M. Schwarz , and I. Daum . 2008. “The Cerebellum Is Involved in Reward‐Based Reversal Learning.” Cerebellum 7: 433–443. 18592331 10.1007/s12311-008-0046-8

[psyp70178-bib-0096] Torriero, S. , M. Oliveri , G. Koch , C. Caltagirone , and L. Petrosini . 2004. “Interference of Left and Right Cerebellar rTMS With Procedural Learning.” Journal of Cognitive Neuroscience 16, no. 9: 1605–1611. 15601522 10.1162/0898929042568488

[psyp70178-bib-0097] Tunc, S. , N. Baginski , J. Lubs , et al. 2019. “Predictive Coding and Adaptive Behavior in Patients With Genetically Determined Cerebellar Ataxia—A Neurophysiology Study.” NeuroImage: Clinical 24: 102043. 31678909 10.1016/j.nicl.2019.102043 PMC6978209

[psyp70178-bib-0098] Ugawa, Y. , B. L. Day , J. C. Rothwell , P. D. Thompson , P. A. Merton , and C. D. Marsden . 1991. “Modulation of Motor Cortical Excitability by Electrical Stimulation Over the Cerebellum in Man.” Journal of Physiology 441, no. 1: 57–72. 1816387 10.1113/jphysiol.1991.sp018738 PMC1180185

[psyp70178-bib-0099] Unger, K. , S. Heintz , and J. Kray . 2012. “Punishment Sensitivity Modulates the Processing of Negative Feedback but Not Error‐Induced Learning.” Frontiers in Human Neuroscience 6: 186. 22754518 10.3389/fnhum.2012.00186 PMC3384291

[psyp70178-bib-0100] van Boxtel, G. J. M. , M. W. van der Molen , and J. R. Jennings . 2005. “Differential Involvement of the Anterior Cingulate Cortex in Performance Monitoring During a Stop‐Signal Task.” Journal of Psychophysiology 19, no. 1: 1–10.

[psyp70178-bib-0101] van Veen, V. , and C. S. Carter . 2002. “The Anterior Cingulate as a Conflict Monitor: fMRI and ERP Studies.” Physiology and Behavior 77, no. 4: 477–482. 12526986 10.1016/s0031-9384(02)00930-7

[psyp70178-bib-0102] Viñas‐Guasch, N. , T. H. B. Ng , J. G. Heng , et al. 2023. “Cerebellar Transcranial Magnetic Stimulation (TMS) Impairs Visual Working Memory.” Cerebellum 22, no. 3: 332–347. 35355219 10.1007/s12311-022-01396-2 PMC9522915

[psyp70178-bib-0103] Wessel, J. R. 2012. “Error Awareness and the Error‐Related Negativity: Evaluating the First Decade of Evidence.” Frontiers in Human Neuroscience 6: 8. 22529791 10.3389/fnhum.2012.00088 PMC3328124

[psyp70178-bib-0104] Wolpert, D. M. , R. C. Miall , and M. Kawato . 1998. “Internal Models in the Cerebellum.” Trends in Cognitive Sciences 2, no. 9: 338–347. 21227230 10.1016/s1364-6613(98)01221-2

[psyp70178-bib-0105] Wu, W. , C. J. Keller , N. C. Rogasch , et al. 2018. “ARTIST: A Fully Automated Artifact Rejection Algorithm for Single‐Pulse TMS‐EEG Data.” Human Brain Mapping 39, no. 4: 1607–1625. 29331054 10.1002/hbm.23938 PMC6866546

[psyp70178-bib-0106] Zhuang, Q. , S. Zhu , X. Yang , et al. 2021. “Oxytocin‐Induced Facilitation of Learning in a Probabilistic Task Is Associated With Reduced Feedback‐ and Error‐Related Negativity Potentials.” Journal of Psychopharmacology (Thousand Oaks, CA) 35, no. 1: 40–49. 10.1177/0269881120972347 33274683

