# Supplementary material for: Cerebellar Single‐Pulse TMS Differentially Affects Early and Late Error Processing in Reinforcement Learning
Source: Psychophysiology. 2025 Oct 31;62(11):e70178. doi: 10.1111/psyp.70178 (PMC12579020; doi:10.1111/psyp.70178)
Supplement: Supplementary file 1 — Data S1: psyp70178‐sup‐0001‐Supinfo.docx. [file PSYP-62-e70178-s001.docx]

**Supplement: Cerebellar single-pulse TMS differentially affects early and late error processing in reinforcement learning**

Dana M. Huvermann^*,1,2^, Adam M. Berlijn^*,1,3,5^, Stefan J. Groiss^3^, Manfred Mittelstaedt^1^, Alfons Schnitzler^3,4^, Christian Bellebaum^1^, Martina Minnerop^3,4,5^, Dagmar Timmann^2^, Jutta Peterburs^1,6^

^1^ Faculty of Mathematics and Natural Sciences, Heinrich Heine University Dusseldorf, Dusseldorf, Germany

^2^Department of Neurology and Center for Translational and Behavioral Neurosciences (C-TNBS), Essen University Hospital, University of Duisburg-Essen, Essen, Germany

^3^Institute of Clinical Neuroscience and Medical Psychology, Medical Faculty & University Hospital Düsseldorf, Heinrich-Heine University Düsseldorf, Düsseldorf, Germany

^4^Department of Neurology, Center for Movement Disorders and Neuromodulation, Medical Faculty, Heinrich-Heine University Düsseldorf, Düsseldorf, Germany

^5^Institute of Neuroscience and Medicine (INM-1), Research Centre Jülich, Jülich, Germany

^6^Institute for Systems Medicine & Department of Human Medicine, MSH Medical School Hamburg, Hamburg, Germany

Corresponding author: [dana.huvermann@hhu.de](mailto:dana.huvermann@hhu.de), +49 211 81-12268

*Shared first authorship

Department of Experimental Psychology

Heinrich-Heine-University Düsseldorf

Universitätsstraße 1, 40225 Düsseldorf, Germany

**Table of Contents**

| Content | Page |
| --- | --- |
| Supplementary Figures | 3 |
| Supplementary Analysis S1  Linear mixed effects analysis of ERN and Pe in patients with cerebellar stroke | 6 |
| Supplementary Analysis S2  Analyses of the influence of cerebellar TMS/stroke on Q_diff_ | 13 |
| Supplementary Analysis S3  Analysis of effects that include TMS timing independently of stimulation site | 24 |
| Supplementary Tables | 26 |
| References | 35 |


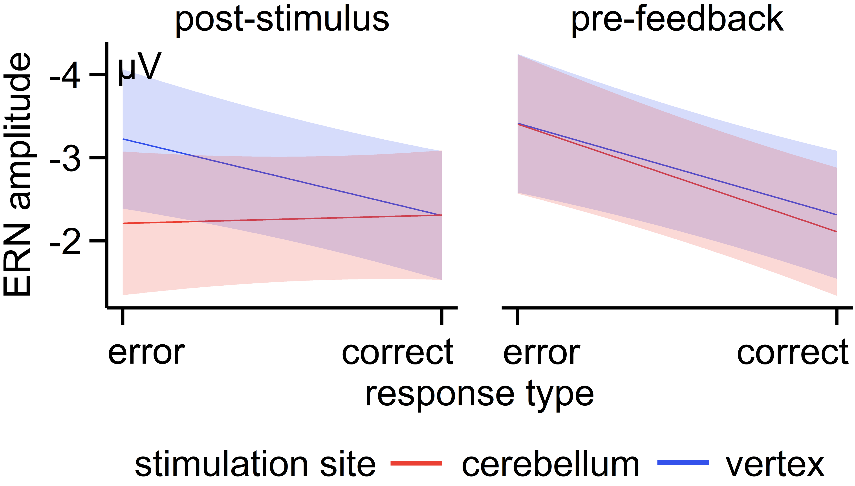


Supplementary Figure S1. Slope estimates for ERN amplitude predicted by response type and modulated by stimulation site and TMS timing. Red lines denote cerebellar stimulation and blue lines vertex stimulation. Coloured bands indicate 95 % confidence intervals. Slope significance was not evaluated as the interaction reached only trend level.


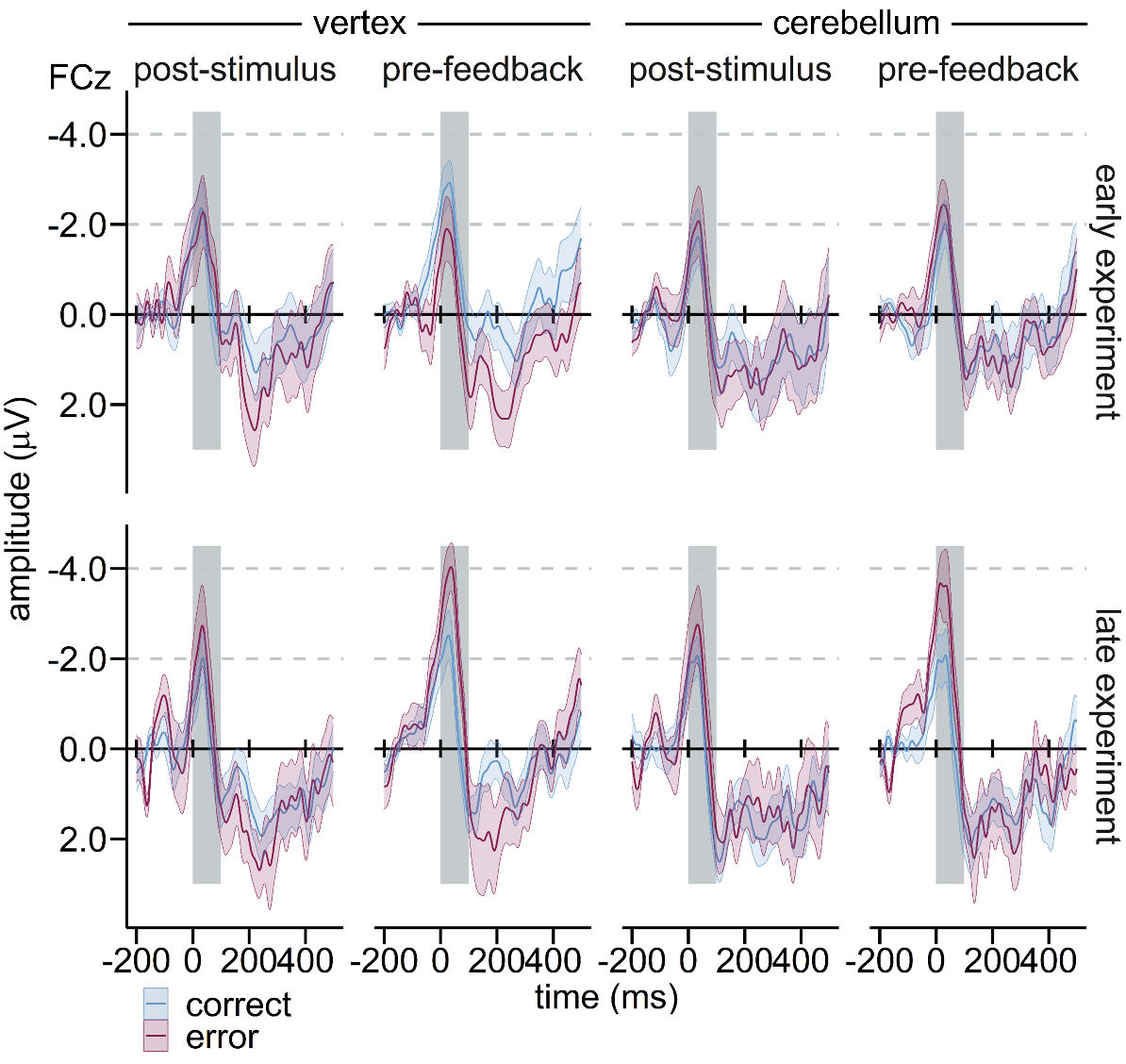


Supplementary Figure S2. Grand-average response-locked ERPs early and late in the task at FCz according to response type (correct, error), stimulation site (cerebellum, vertex), and TMS timing (post-stimulus, pre-feedback). Blue lines denote correct responses, red lines errors. Coloured bands display standard errors.


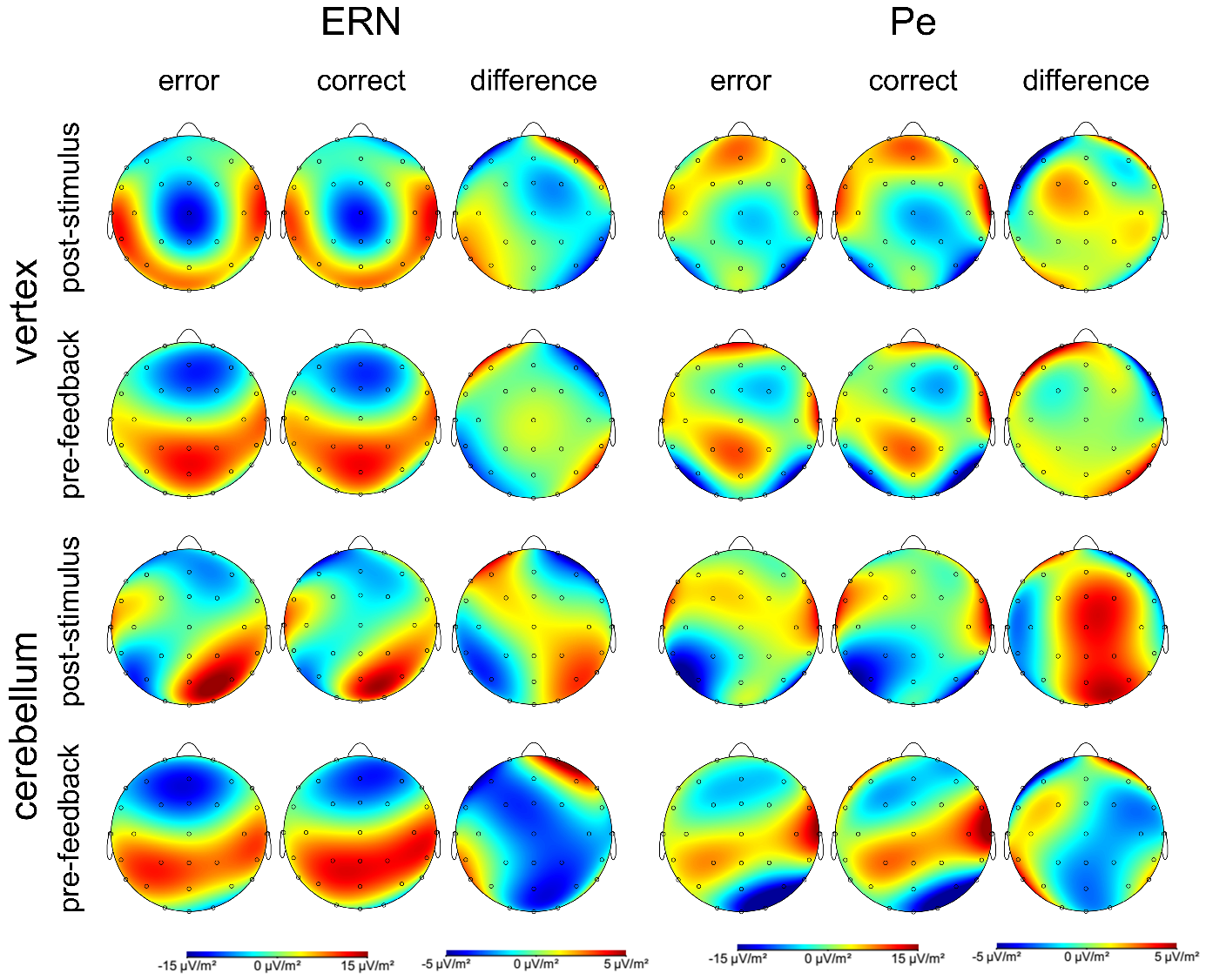


Supplementary Figure S3. Topographical plots for the response-locked ERP for the time window between response onset and 100 ms post-response (ERN) and between 200 and 400 ms post-response (Pe) for errors, correct responses, and the difference signal (errors – correct responses) as a function of stimulation site (cerebellum, vertex) and TMS timing (post-stimulus, pre-feedback).

**Supplementary Analysis S1: LME analysis of ERN and Pe in patients with cerebellar stroke**

***Method***

Note that the present study was part of a larger investigation of cerebellar contributions to reinforcement learning and presents novel follow-up analyses of data reported in a previous study by our group (1) that focused on outcome/feedback processing and thus did not analyse response-locked ERPs. There, we performed two parallel experiments in both 1) patients with chronic cerebellar stroke and matched controls and 2) young healthy adults receiving cerebellar and vertex (control) TMS in separate sessions. Both studies used similar probabilistic feedback learning paradigms as reported in the main text. The experimental protocol was defined prior to experiments and preregistered to OSF (https://osf.io/rd3xb). Raw data and code used for preprocessing and analysis is openly available at <https://osf.io/cqf97>.

*Participants*

Sample characteristics are detailed in Huvermann et al. (1). Data from 26 patients with post-acute stroke isolated to the cerebellum and 26 controls entered the analysis. Controls were matched for age, sex, years of education, Edinburgh Handedness Inventory (2), Mehrfachwahl-Wortschatz-Test-B (3), and Beck Depression Inventory-II score (4). Patients and controls were mostly middle-aged (M_patients_ = 56.2, SD_patients_ = 12.1; M_controls_ = 56.4, SD_controls_ = 12.7), male (patients: 20 men, 6 women; controls: 19 men, 7 women), and right-handed according to EHI (patients: 19 right-handed, 3 left-handed, 4 ambidextrous; controls: 21 right-handed, 1 left-handed, 4 ambidextrous). Patients (*M* = 1.73, *SD* = 1.78) had a higher score on the scale for the assessment and rating of ataxia (SARA, 5) than control participants (*M* = 0.46, *SD* = 1.51), *t*(47.01) = 2.74, *p* = .009.

*Procedure*

Please see Huvermann et al. (1) for a detailed description. Experiments took place on two (usually consecutive) days. On both days, an EEG task as described for the TMS experiment was conducted. However, no stimulation took place. One day included a task version with short feedback delay as described for the TMS experiment, while the other day included a long feedback delay (6500 ms). Additionally, some adaptations were made to optimise the task for the older sample, i.e., extended response window (3000 ms instead of 1000 ms) and increased feedback contingency (90 % instead of 80 %).

*EEG recording and preprocessing*

Data were recorded at 1,000 Hz from 28 active Ag/AgCl electrodes positioned according to the 10-20 system (6), using the BrainAmp amplifier and BrainVision Recorder 1.21 (Brain Products GmbH, Gilching, Germany). Impedances were kept below 25 kΩ.

Preprocessing was performed using BrainVision Analyzer 2 (version 2.2, Brain Products GmbH, Gilching, Germany). First, a DC detrend was applied and data were bandpass and notch filtered (1-30 Hz, 50 Hz). Eye movements were removed using Ocular Correction ICA, and data were subsequently segmented around the response markers, starting 200 ms preceding response onset and ending 600 ms post-response. Only trials with contingent feedback were analysed. Data were baseline-corrected based on the time window 200-100 ms pre-response, followed by an automatic artefact rejection. On average, 1.05 % of segments were rejected (*SD* = 2.17 % of segments). Data were then averaged and exported according to response type and feedback delay. See Huvermann et al. (1) for further details.

Peak detection was performed in Matlab as described for the TMS study in the main manuscript (Methods section).

*Statistical data analysis*

Data analysis was conducted as described for the TMS study in the main manuscript.

**ERN and Pe.** The same approach was applied for ERN and Pe. LME analysis was performed with the categorical fixed effects response type (-0.5: error, 0.5: correct), group (-0.5: controls, 0.5: patients), feedback delay (-0.5: short delay, 0.5: long delay), and the continuous factor trial number. We also included all interactions of these factors as fixed effects. One participant for ERN and 5 participants for Pe had to be excluded due to exceeding the Cook’s distance criterion. The model equations were as follows:

$$ERN \sim1+response type*feedback delay*group*trial number + (1 | subject)$$

$$Pe \sim1+response type*feedback delay*group*trial number +$$

$$(1+response type:feedback delay:trial number | subject)$$

***Results***

*Accuracy*

As reported in Huvermann et al. (1), no differences between patients and controls emerged for learning. A general effect of block was found, indicative of a general learning effect. On average, 6.9 errors per block, feedback delay, and participant were commited (*SD* = 4.3 errors).

*ERN – response type*

Grand Averages for the response-locked ERPs at FCz for correct responses and errors according to group, feedback delay, and trial number (early, late experiment) can be found in Supplementary Figure S4A.

We found an interaction between feedback delay and group (β = 0.52, *SE* = 0.19, *t*(15955.01) = 2.74, *p* = .006; see Supplementary Figure S4B). While for controls, there was no difference in ERN between short and long feedback delay conditions (β = -0.12, *SE* = 0.13, *t* = 0.92, *p* = .718), the ERN was increased in patients for short over long feedback delays (β = 0.37, *SE* = 0.13, *t* = 2.91, *p* = .007).

Complete inferential statistics can be found in Supplementary Table S3.


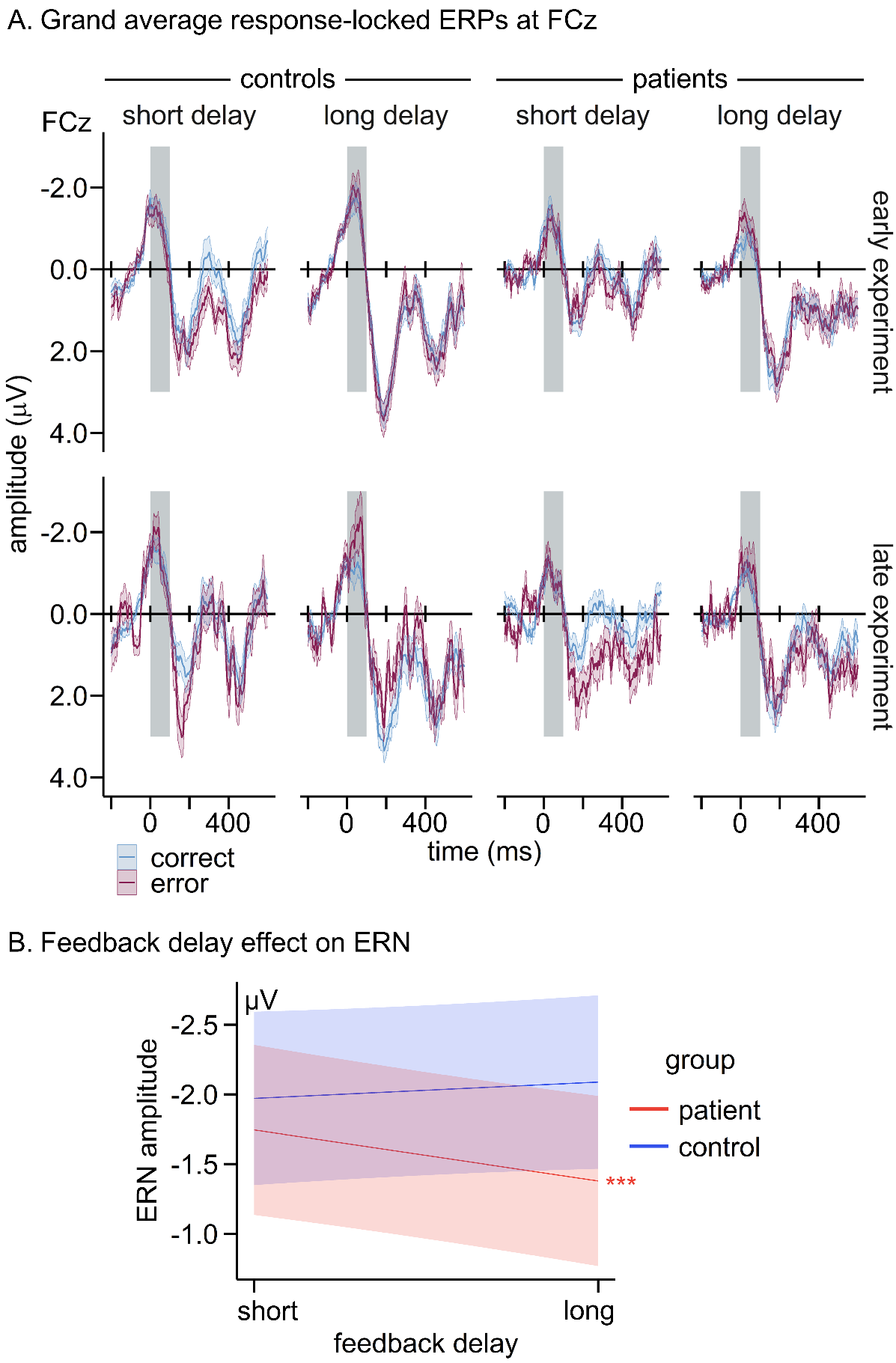
Supplementary Figure S4. A) Grand-average response-locked ERPs early and late in the task at FCz according to response type (correct, error), group (controls, patients) and feedback delay (short, long). Blue lines denote correct responses, red lines errors. Coloured bands display standard errors. B) Slope estimates for ERN amplitude predicted by feedback delay and modulated by group. Red lines denote patients and blue lines controls. Coloured bands indicate 95 % confidence intervals. * *p* < .05. ** *p* < .01. *** *p* < .001. n_error_ = 5578, n_correct_ = 10436.

*Pe – response type*

Grand Averages for the response-locked ERPs at Pz for correct responses and errors according to group, feedback delay, and trial number (early, late experiment) can be found in Supplementary Figure S5A.

The Pe was increased for errors relative to correct responses (β = -0.53, *SE* = 0.10, *t*(14737.43) = 5.40, *p* < .001), for long compared to short feedback delay (β = 1.02, *SE* = 0.09, *t*(14197.69) = 10.83, *p* < .001), and decreased across the course of the task (β = -0.26, *SE* = 0.05, *t*(13716.24) = 5.63, *p* < .001). We also found an interaction between group and trial number (β = 0.23, *SE* = 0.09, *t*(13716.24) = 2.48, *p* = .013; see Supplementary Figure S5B). The Pe decreased more strongly across the course of the task for controls (β = -0.37, *SE* = 0.06, *t* = 5.93, *p* < .001), but only at trend level for patients (β = -0.14, *SE* = 0.06, *t* = 2.12, *p* = .069).

Complete inferential statistics can be found in Supplementary Table S4.


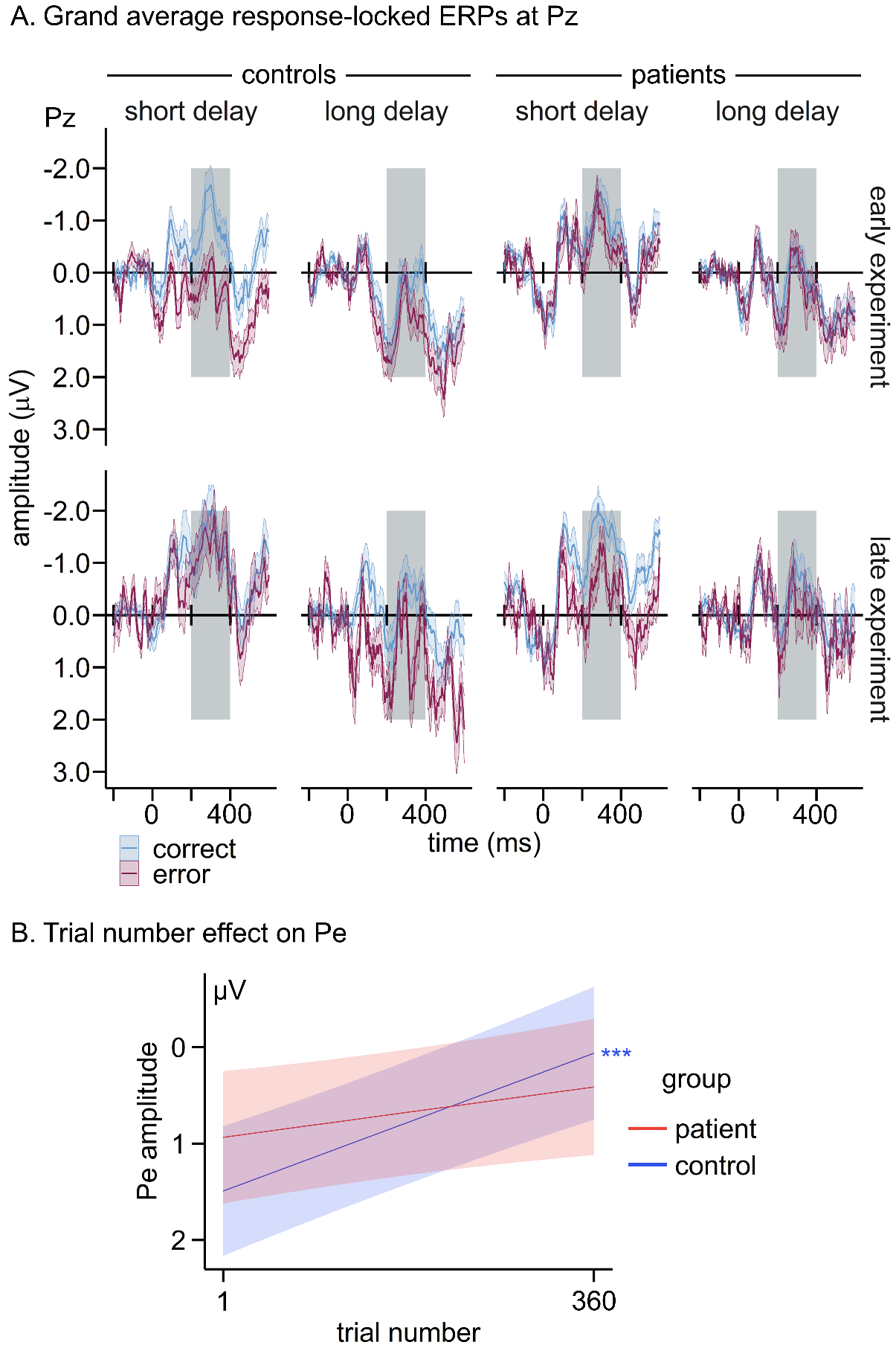


Supplementary Figure S5. A) Grand-average response-locked ERPs early and late in the task at Pz according to response type (correct, error), group (controls, patients) and feedback delay (short, long). Blue lines denote correct responses, red lines errors. Coloured bands display standard errors. B) Slope estimates for Pe amplitude predicted by trial number and modulated by group. Red lines denote patients and blue lines controls. Coloured bands indicate 95 % confidence intervals. * *p* < .05. ** *p* < .01. *** *p* < .001. n_error_ = 5238, n_correct_ = 9520.

**Supplementary Analysis S2: Analyses of the influence of cerebellar TMS/stroke on Q_diff_**

***Methods***

*Modelling of action values*

Action values in each trial were estimated based on a reinforcement learning model (7), following previous studies (8–13). This approach has been shown to be highly correlated with the gold standard (i.e., subjective ratings, 10). Please see Huvermann et al. (1) for details.

We then calculated the relative subjective value of the chosen option as the difference between the Q-value of the chosen outcome minus the Q-value of the unchosen option, Q_diff_. Relative action values have been shown to be more reliable than absolute action values (14). A high positive Q_diff_ expresses that the action perceived as better was chosen, while a negative Q_diff_ expresses that the action perceived as worse was chosen, which would indicate an error. A Q_diff_ around 0 thus corresponds to a high response conflict. The code used to model and simulate behaviour is provided in Huvermann et al. (1).

*Data analysis – TMS study*

For the additional analysis of Q_diff_, the same analysis design was applied, replacing the binary predictor response type with the continuous predictor Q_diff_. No participants for ERN and one participant for Pe had to be excluded due to exceeding the Cook’s distance criterion. Model equations were as follows:

$$ERN/Pe \sim1+Q_{diff}*stimulation site*TMS timing*trial number +$$

$$(1+Q_{diff}:stimulation site:TMS timing:trial number | subject)$$

*Data analysis – patient study*

For the additional analysis of Q_diff_, the same analysis design was applied, again replacing response type with Q_diff_. One participant for ERN and 11 participants for Pe had to be excluded due to exceeding the Cook’s distance criterion. Model equations were as follows:

$$ERN/Pe \sim1+Q_{diff}*feedback delay*group*trial number +$$

$$(1+Q_{diff}:feedback delay:trial number | subject)$$

***Results***

*ERN – TMS study*

Grand Averages for the response-locked ERPs at FCz for negative, neutral and positive Q_diff_ according to stimulation site, TMS timing and trial number (early, late experiment) can be found in Supplementary Figure S6A.

We found a higher (i.e., more negative) ERN with decreasing Q_diff_ values (β = 0.43, *SE* = 0.08, *t*(4725.26) = 5.16, *p* < .001). This effect was modulated by trial number (β = 0.24, *SE* = 0.08, *t*(958.20) = 2.84, *p* = .005). Follow-up simple slope analyses showed that Q_diff_ significantly affected the ERN late in the experiment (β = 0.66, *SE* = 0.11, *t* = 5.91, *p* < .001) but only on trend-level early in the experiment (β = 0.24, *SE* = 0.12, *t* = 2.01, *p* = .089).

Importantly, we found an interaction between Q_diff_, trial number, and stimulation site (β = -0.40, *SE* = 0.17, *t*(984.23) = -2.44, *p* = .015; see Supplementary Figure S6B). Early in the task, Q_diff_ was again not reflected in ERN for both stimulation sites (both *p* ≥ .355). Late in the task, ERN was increased with decreasing Q_diff_ for vertex TMS (β = 1.01, *SE* = 0.16, *t* = 6.24, *p* < .001) but not for cerebellar TMS (β = 0.28, *SE* = 0.15, *t* = 1.87, *p* = .246).

The complete inferential pattern can be found in Supplementary Table S5. Effects including the TMS timing factor independently from stimulation site are reported in Supplementary Analysis S3.


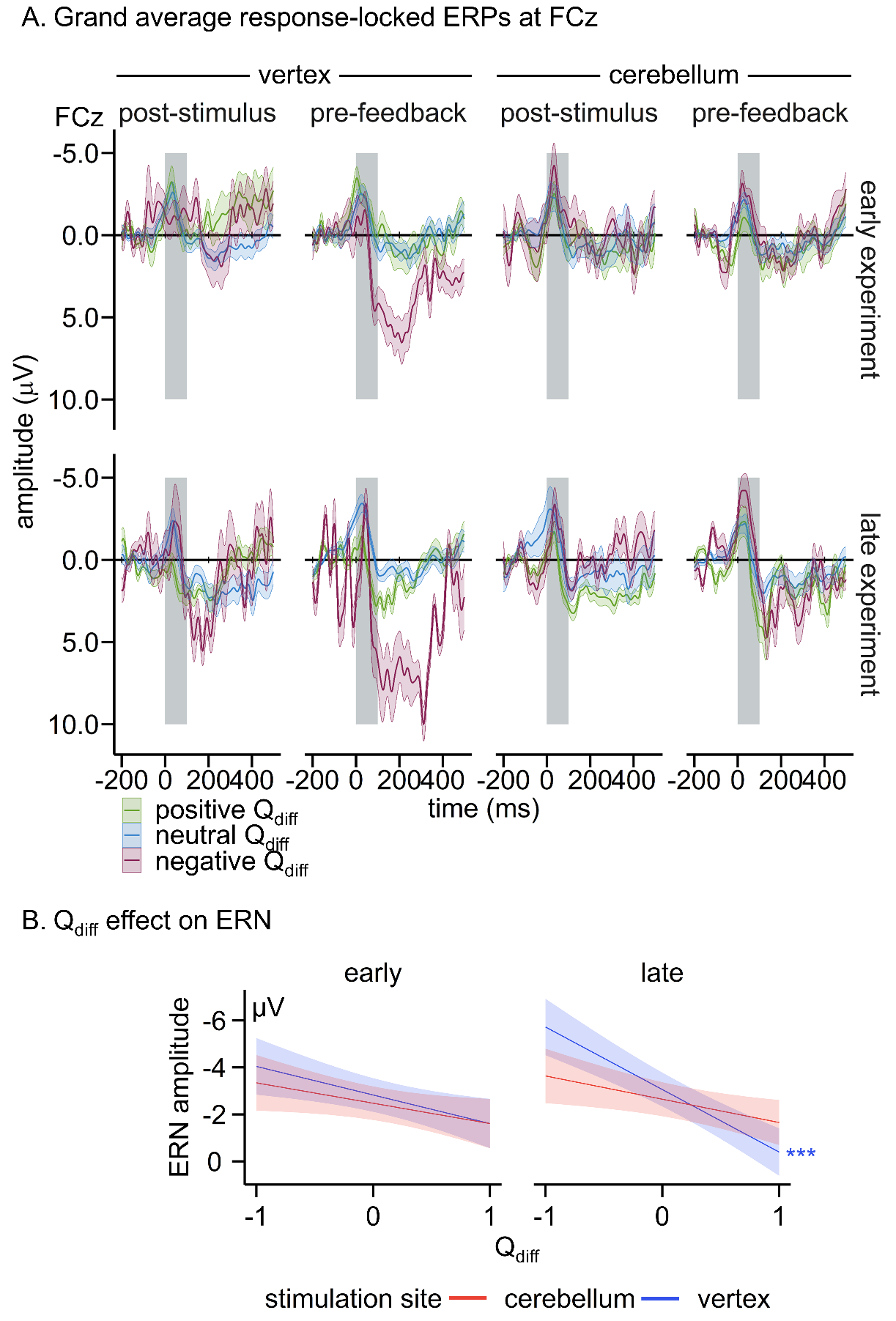
Supplementary Figure S6. A) Grand-average response-locked ERPs early and late in the task at FCz according to Q_diff_ bins (positive: >0.3, neutral: ≥ -0.3 and ≤ 0.3, negative: < -0.3), stimulation site (cerebellum, vertex) and TMS timing (post-stimulus, pre-feedback). Green lines denote positive Q_diff_, blue lines neutral Q_diff_ and red lines negative Q_diff_. Coloured bands display standard errors. B) Slope estimates for ERN amplitude predicted by Q_diff_ and modulated by stimulation site and trial number (early, late experiment). Red lines denote cerebellar stimulation and blue lines vertex stimulation. Coloured bands indicate 95 % confidence intervals. * p < .05. ** p < .01. *** p < .001. n_error_ = 2702, n_correct_ = 4777.

*Pe – TMS study*

Grand Averages for the response-locked ERPs at Pz for negative, neutral and positive Q_diff_ according to stimulation site, TMS timing and trial number (early, late experiment) can be found in Supplementary Figure S7A.

We found an increased Pe with decreasing Q_diff_ values (β = -0.56, *SE* = 0.08, *t*(5816.27) = 7.16, *p* < .001) and across the course of the experiment (β = 0.24, *SE* = 0.07, *t*(6325.84) = 3.43, *p* = .001). Q_diff_ interacted with stimulation site (β = -0.42, *SE* = 0.15, *t*(6808.80) = 2.70, *p* = .007), such that Q_diff_ was reflected in Pe more strongly for cerebellar TMS (β = -0.78, *SE* = 0.11, *t* = 7.21, *p* < .001) than vertex TMS (β = -0.35, *SE* = 0.11, *t* = 3.19, *p* = .003).

Importantly, Q_diff_ further interacted with stimulation site and TMS timing (β = 1.30, *SE* = 0.31, *t*(6933.62) = 4.24, *p* < .001; see Supplementary Figure S7B). For vertex TMS, Q_diff_ was reflected in Pe for pre-feedback TMS (β = -0.47, *SE* = 0.17, *t* = 2.68, *p* = .029) but not for post-stimulus TMS (β = -0.23, *SE* = 0.13, *t* = 1.82, *p* = .273). For cerebellar TMS, Q_diff_ was reflected strongly in Pe for post-stimulus TMS (β = -1.29, *SE* = 0.16, *t* = 8.16, *p* < .001) but not pre-feedback TMS (β = -0.27, *SE* = 0.15, *t* = 1.85, *p* = .257).

The complete inferential pattern can be found in Supplementary Table S6. Effects including the TMS timing factor independently from stimulation site are reported in Supplementary Analysis S3.


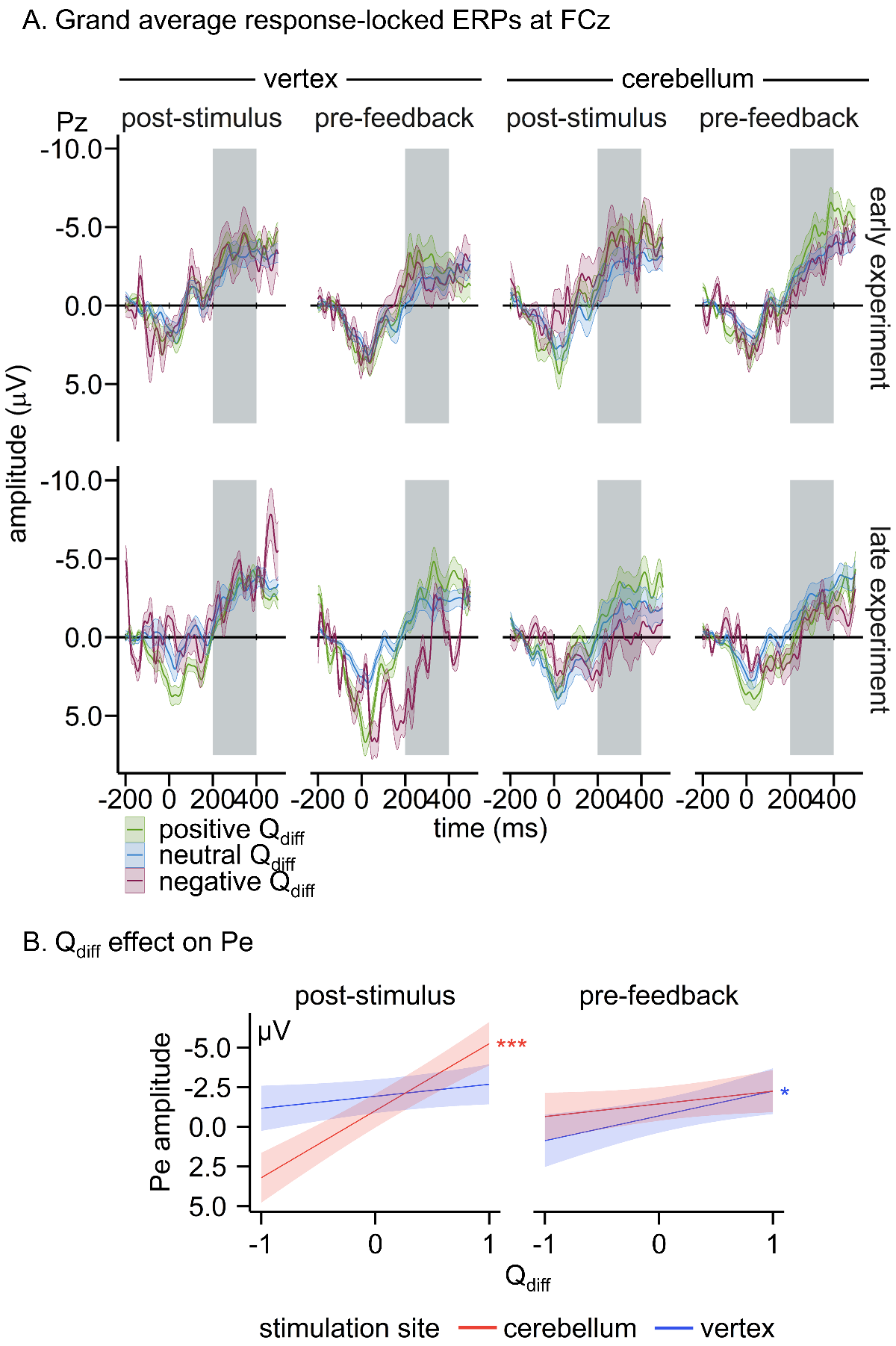


Supplementary Figure S7. A) Grand-average response-locked ERPs early and late in the task at Pz according to Q_diff_ bins (positive: >0.3, neutral: ≥ -0.3 and ≤ 0.3, negative: < -0.3), stimulation site (cerebellum, vertex) and TMS timing (post-stimulus, pre-feedback). Green lines denote positive Q_diff_, blue lines neutral Q_diff_ and red lines negative Q_diff_. Coloured bands display standard errors. B) Slope estimates for Pe amplitude predicted by Q_diff_ and modulated by stimulation site and TMS timing. Red lines denote cerebellar stimulation and blue lines vertex stimulation. Coloured bands indicate 95 % confidence intervals. * p < .05. ** p < .01. *** p < .001. n_error_ = 2607, n_correct_ = 4957.

*ERN – patient study*

Grand Averages for the response-locked ERPs at FCz for negative, neutral, and positive Q_diff_ according to group, feedback delay, and trial number (early, late experiment) are provided in Supplementary Figure S8A.

We found an interaction between feedback delay and group (β = 0.45, *SE* = 0.18, *t*(13665.62) = 2.49, *p* = .013). While the ERN was increased for short relative to long feedback delays in patients (β = 0.36, *SE* = 0.13, *t* = 2.87, *p* = .008), this was not the case in controls (β = -0.09, *SE* = 0.13, *t* = 0.68, *p* = .988).

Additionally, the interaction between Q_diff_, feedback delay, and group was significant (β = -0.46, *SE* = 0.19, *t*(11988.66) = 2.46, *p* = .014; see Supplementary Figure S8B). However, resolving it via simple slope analysis with Q_diff_ as a predictor, none of the slopes reached significance (all *p* ≥ .204), except for a trend-level effect of Q_diff_ for patients and long-feedback delays (β = -0.23, *SE* = 0.10, *t* = 2.26, *p* = .096).

Complete inferential statistics can be found in Supplementary Table S7.


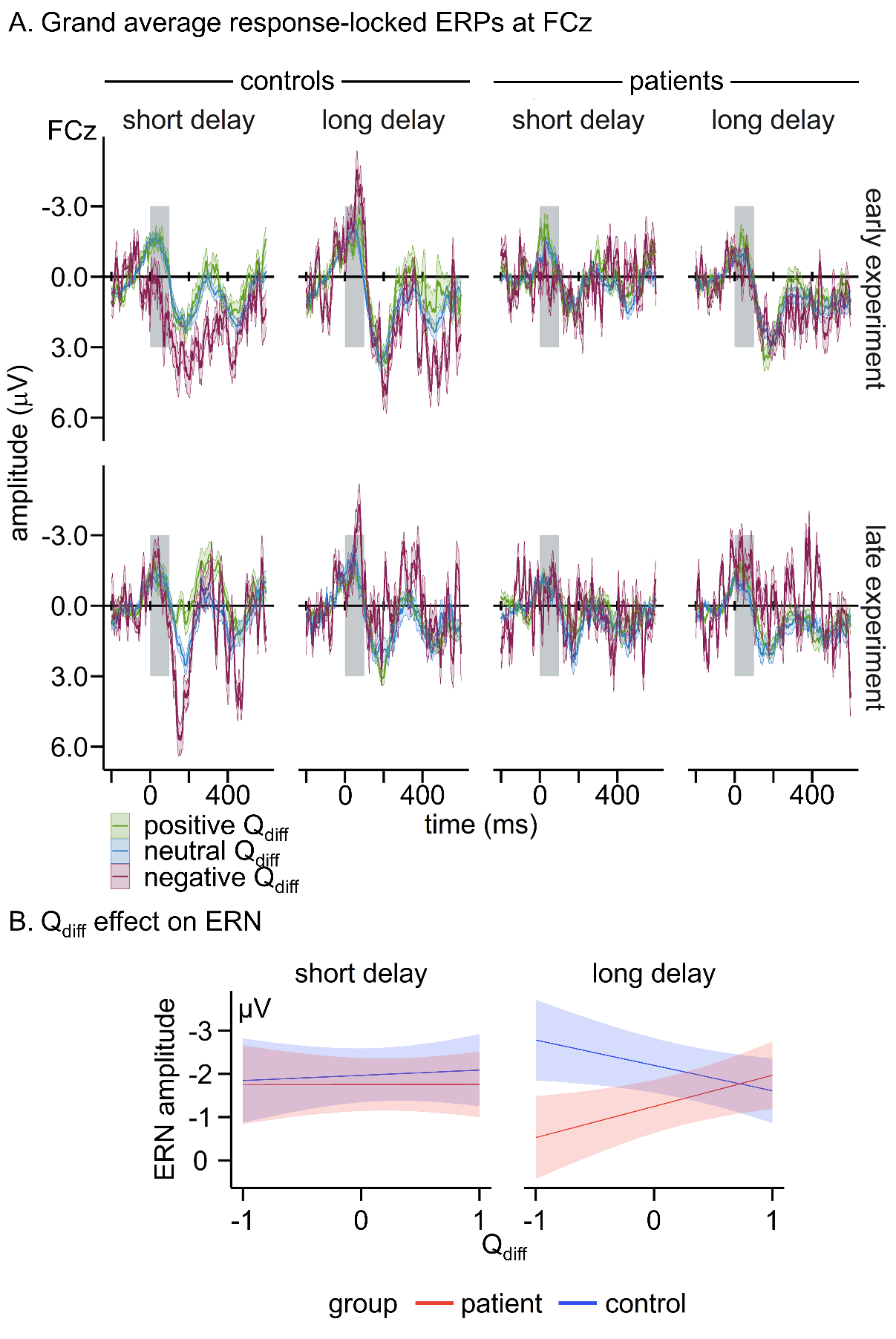
Supplementary Figure S8. A) Grand-average response-locked ERPs early and late in the task at FCz according to Q_diff_ bins (positive: >0.3, neutral: ≥ -0.3 and ≤ 0.3, negative: < -0.3), group (controls, patients) and feedback delay (short, long). Blue lines denote correct responses, red lines errors. Coloured bands display standard errors. B) Slope estimates for ERN amplitude predicted by Q_diff_ and modulated by group and feedback delay. Red lines denote patients and blue lines controls. Coloured bands indicate 95 % confidence intervals. * *p* < .05. ** *p* < .01. *** *p* < .001. n_error_ = 5578, n_correct_ = 10436.

*Pe – patient study*

Grand Averages for the response-locked ERPs at Pz for negative, neutral, and positive Q_diff_ according to group, feedback delay, and trial number (early, late experiment) can be found in Supplementary Figure S9A.

The Pe increased with decreasing Q_diff_ (β = -0.49, *SE* = 0.06, *t*(7728.01) = 7.58, *p* < .001), and was more pronounced for long compared to short feedback delay (β = 1.13, *SE* = 0.10, *t*(11258.39) = 11.39, *p* < .001), and decreased over the course of the task (β = -0.27, *SE* = 0.05, *t*(6318.58) = 5.45, *p* < .001).

An interaction between Q_diff_ and trial number emerged (β = -0.12, *SE* = 0.06, *t*(2877.32) = 2.12, *p* = .034; see Supplementary Figure S9B). Q_diff_ was more strongly reflected in the Pe late in the task (β = -0.59, *SE* = 0.09, *t* = 6.81, *p* < .001) compared to early in the task (β = -0.38, *SE* = 0.08, *t* = 4.64, *p* < .001).

An interaction between group and trial number emerged (β = 0.32, *SE* = 0.10, *t*(6318.58) = 3.25, *p* = .001; see Supplementary Figure S9C). While for controls, the Pe decreased over the course of the task (β = -0.41, *SE* = 0.07, *t* = 6.04, *p* < .001), this was not the case for patients (β = -0.10, *SE* = 0.07, *t* = 1.47, *p* = .285).

An interaction between Q_diff_, feedback delay, and group emerged (β = -0.58, *SE* = 0.23, *t*(11319.05) = 2.54, *p* = .011; see Supplementary Figure S9D). For controls, Q_diff_ was reflected in the Pe for both short (β = -0.68, *SE* = 0.14, *t* = 5.02, *p* < .001) and long feedback delays (β = -0.43, *SE* = 0.10, *t* = 4.21, *p* < .001). For patients, Q_diff_ was reflected in the Pe for long (β = -0.57, *SE* = 0.13, *t* = 4.51, *p* < .001) but not short feedback delay (β = -0.26, *SE* = 0.12, *t* = 2.26, *p* = .095).

Complete inferential statistics can be found in Supplementary Table S8.


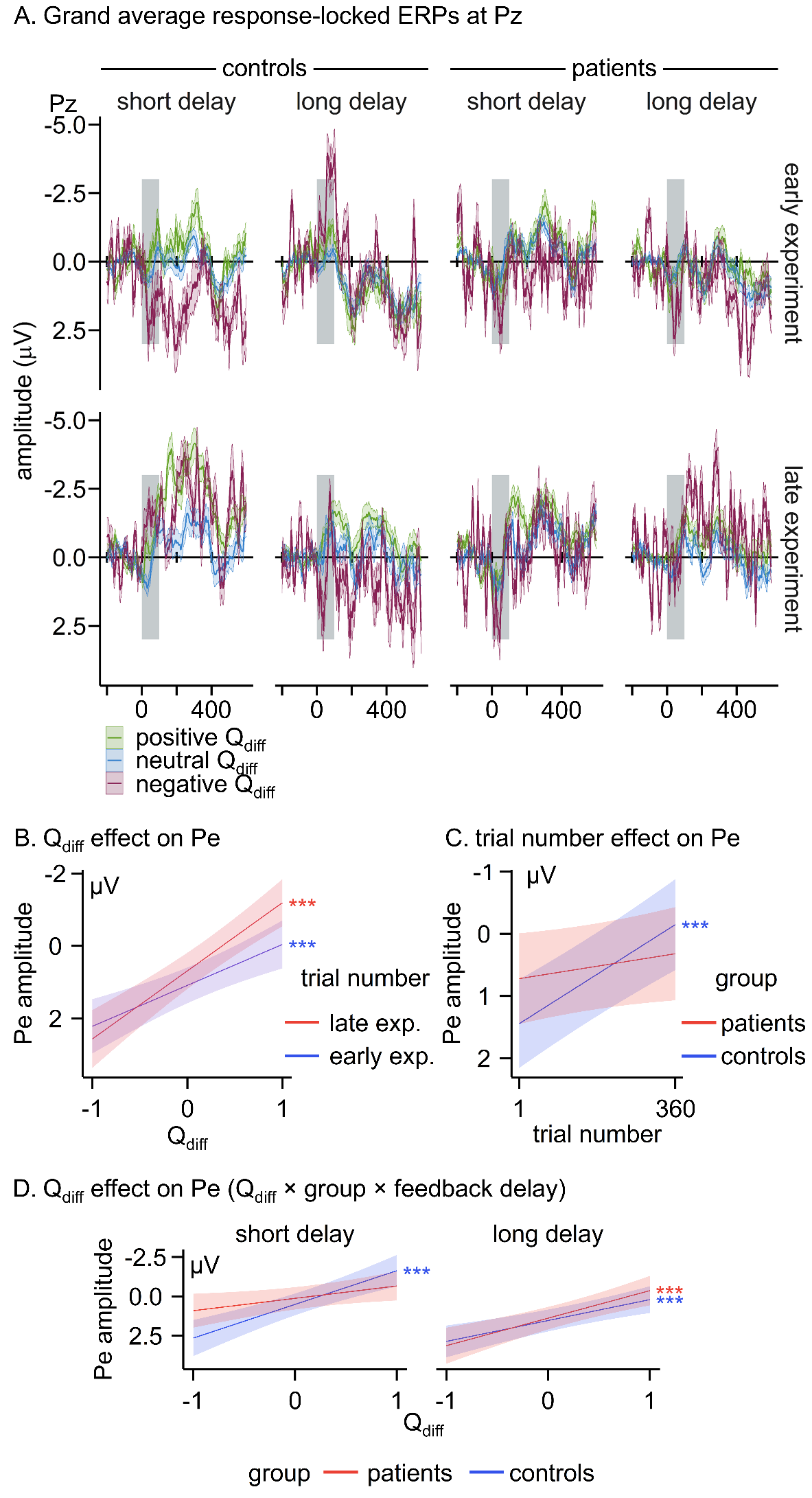


Supplementary Figure S9. A) Grand-average response-locked ERPs early and late in the task at Pz according to Q_diff_ bins (positive: >0.3, neutral: ≥ -0.3 and ≤ 0.3, negative: < -0.3), group (controls, patients) and feedback delay (short, long). Blue lines denote correct responses, red lines errors. Coloured bands display standard errors. B) Slope estimates for Pe amplitude predicted by Q_diff_ and modulated by trial number (early, late experiment). Red lines denote late experiment and blue lines early experiment. C) Slope estimates for Pe amplitude predicted by trial number and modulated by group. Red lines denote patients and blue lines controls. D) Slope estimates for Pe amplitude predicted by Q_diff_ and modulated by group and feedback delay. Red lines denote patients and blue lines controls. Coloured bands indicate 95 % confidence intervals. * *p* < .05. ** *p* < .01. *** *p* < .001. n_error_ = 4909, n_correct_ = 7984.

**Supplementary Analysis S3: Analysis of effects that include TMS timing independently of stimulation site**

***ERN – response type***

A significant interaction between response type and TMS timing emerged (β = 0.79, *SE* = 0.32, *t*(7450.32) = 2.45, *p* = .014). The ERN was increased for errors over correct responses for pre-feedback TMS (β = 1.19, *SE* = 0.22, *t* = 5.33, *p* < .001) but not post-stimulus TMS (β = 0.42, *SE* = 0.23, *t* = 1.80, *p* = .143).

***ERN – Q_diff_***


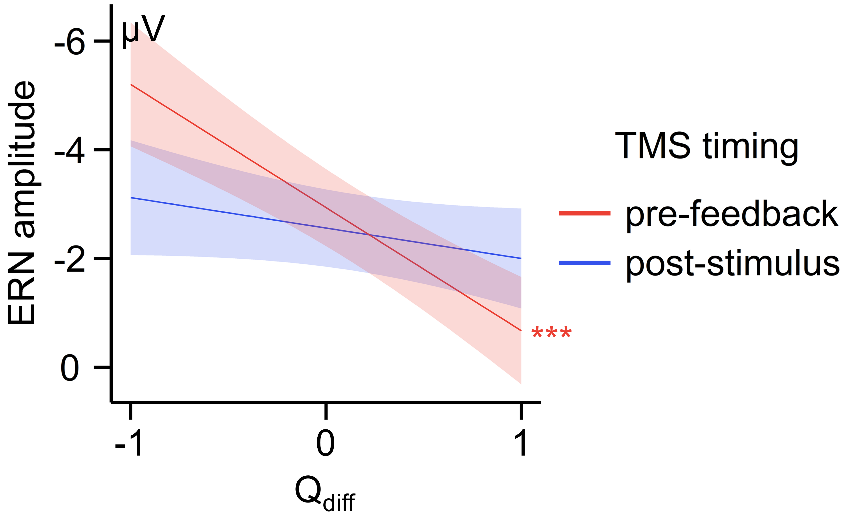
A significant interaction between response type and TMS timing emerged (β = 0.52, *SE* = 0.16, *t*(7163.28) = 3.23, *p* = .001; see Supplementary Figure S10). The ERN was increased for low Q_diff_ values for pre-feedback TMS (β = 0.69, *SE* = 0.12, *t* = 5.55, *p* < .001) but not post-stimulus TMS (β = 0.17, *SE* = 0.11, *t* = 1.57, *p* = .232).

Supplementary Figure S10. Slope estimates for ERN amplitude predicted by Q_diff_ and modulated by TMS timing. Red line denotes pre-feedback stimulation and blue line post-stimulus stimulation. Coloured bands indicate 95 % confidence intervals. * p < .05. ** p < .01. *** p < .001.

***Pe – response type***

An interaction between response type and TMS timing emerged (β = 1.00, *SE* = 0.29, *t*(7850.81) = 3.44, *p* = .001). Pe was increased for errors over correct responses more strongly for post-stimulus TMS (β = -1.50, *SE* = 0.21, *t* = 7.16, *p* < .001) over pre-feedback TMS (β = -0.49, *SE* = 0.21, *t* = 2.35, *p* = .037).

An interaction between stimulation site and TMS timing emerged (β = -1.53, *SE* = 0.29, *t*(7826.63) = 5.30, *p* < .001). For post-stimulus TMS, Pe was increased for cerebellar over vertex TMS (β = 0.60, *SE* = 0.19, *t* = 3.06, *p* = .004). For pre-feedback TMS, the pattern was reversed, with Pe decreased for cerebellar over vertex TMS (β = -0.62, *SE* = 0.19, *t* = 3.20, *p* = .003).

***Pe – Q_diff_***

We found a decreased Pe for post-stimulus relative to pre-feedback stimulation (β = 0.60, *SE* = 0.14, *t*(7347.09) = 4.23, *p* < .001).

An interaction between Q_diff_ and TMS timing emerged (β = 0.40, *SE* = 0.15, *t*(7323.77) = 2.64, *p* = .008). The Pe was increased for errors over correct responses more strongly for post-stimulus TMS (β = -0.76, *SE* = 0.10, *t* = 7.45, *p* < .001) over pre-feedback TMS (β = -0.37, *SE* = 0.11, *t* = 3.23, *p* = .003).

An interaction between stimulation site and TMS timing emerged (β = -1.04, *SE* = 0.28, *t*(7250.07) = 3.70, *p* < .001). For post-stimulus TMS, the Pe was increased for cerebellar relative to vertex TMS at trend-level (β = 0.41, *SE* = 0.20, *t* = 2.05, *p* = .080). For pre-feedback TMS, the pattern was reversed, with decreased Pe amplitudes for cerebellar over vertex TMS (β = -0.65, *SE* = 0.20, *t* = 3.30, *p* = .002).

| Supplementary Table S1: Inferential statistics for the LME analysis examining the effect of response type, stimulation site, TMS timing, and trial number on ERN amplitudes in the TMS study | | | | | |
| --- | --- | --- | --- | --- | --- |
| Fixed Effects | | | | | |
|  | *Est*/β | *SE* | *df* | *t* | *p* |
| **(Intercept)** | **-2.66** | **0.35** | **23.16** | **-7.53** | **< .001** |
| **response type** | **0.81** | **0.16** | **7451.23** | **4.94** | **< .001** |
| stimulation site | 0.31 | 0.16 | 7416.28 | 1.92 | .055 |
| TMS timing | -0.30 | 0.16 | 7416.99 | -1.87 | .061 |
| trial number | -0.15 | 0.08 | 6758.03 | -1.90 | .057 |
| response type × stimulation site | -0.42 | 0.32 | 7447.26 | -1.30 | .194 |
| **response type × TMS timing** | **0.79** | **0.32** | **7450.32** | **2.45** | **.014** |
| stimulation site × TMS timing | -0.40 | 0.32 | 7359.83 | -1.24 | .214 |
| **response type × trial number** | **0.51** | **0.16** | **7394.78** | **3.22** | **.001** |
| stimulation site × trial number | 0.01 | 0.16 | 6753.12 | 0.06 | .956 |
| TMS timing × trial number | -0.22 | 0.16 | 7082.67 | -1.39 | .165 |
| response type × stimulation site × TMS timing | 1.22 | 0.65 | 7448.34 | 1.88 | .060 |
| **response type × stimulation site × trial number** | **-0.80** | **0.32** | **7431.03** | **-2.53** | **.012** |
| response type × TMS timing × trial number | 0.40 | 0.32 | 7319.76 | 1.27 | .204 |
| stimulation site × TMS timing × trial number | 0.23 | 0.32 | 4939.87 | 0.71 | .480 |
| response type × stimulation site × TMS timing × trial number | 0.22 | 0.76 | 23.94 | 0.29 | .774 |
| Random Effects | | | | | |
|  | *Variance* | *SD* | *Corr* | | |
| subject (Intercept) | 2.85 | 1.69 |  |  |  |
| subject (response type × stimulation site × TMS timing × trial number) | 4.18 | 2.04 | -0.33 |  |  |
| Residual | 42.64 | 6.53 |  |  |  |
| Model fit | | | | | |
|  | marginal | | conditional | | |
| R^2^ | 0.01 | | 0.07 | | |
| Key: *p*-values for fixed effects calculated using Satterthwaites approximations. Model equation: ERN ~ 1 + response type*stimulation site*TMS timing*trial number + (1 + response type:stimulation site:TMS timing:trial number \| subject) | | | | | |
| *Note*. *n*_subjects_ = 24, *n*_observations_ = 7479. Bold text indicates significant effects. | | | | | |
| Supplementary Table S2: Inferential statistics for the LME analysis examining the effect of response type, stimulation site, TMS timing, and trial number on Pe amplitudes in the TMS study | | | | | |
| Fixed Effects | | | | | |
|  | *Est*/β | *SE* | *df* | *t* | *p* |
| **(Intercept)** | **-1.24** | **0.54** | **23.07** | **-2.31** | **.030** |
| **response type** | **-0.99** | **0.15** | **7848.81** | **-6.68** | **< .001** |
| stimulation site | 0.04 | 0.14 | 7843.79 | 0.29 | .770 |
| TMS timing | 0.17 | 0.14 | 7844.56 | 1.19 | .235 |
| **trial number** | **0.24** | **0.07** | **7734.12** | **3.37** | **.001** |
| response type × stimulation site | -0.46 | 0.29 | 7840.26 | -1.59 | .112 |
| **response type × TMS timing** | **1.00** | **0.29** | **7850.81** | **3.44** | **.001** |
| **stimulation site × TMS timing** | **-1.53** | **0.29** | **7826.63** | **-5.30** | **< .001** |
| response type × trial number | -0.21 | 0.15 | 7844.66 | -1.47 | .141 |
| stimulation site × trial number | 0.02 | 0.15 | 7678.77 | 0.12 | .903 |
| TMS timing × trial number | 0.01 | 0.14 | 7788.22 | 0.09 | .930 |
| **response type × stimulation site × TMS timing** | **2.07** | **0.58** | **7855.11** | **3.55** | **< .001** |
| response type × stimulation site × trial number | 0.21 | 0.29 | 7841.88 | 0.71 | .478 |
| response type × TMS timing × trial number | 0.33 | 0.29 | 7850.33 | 1.14 | .256 |
| stimulation site × TMS timing × trial number | 0.16 | 0.29 | 6843.02 | 0.56 | .574 |
| response type × stimulation site × TMS timing × trial number | 1.46 | 0.83 | 24.63 | 1.76 | .090 |
| Random Effects | | | | | |
|  | *Variance* | *SD* | *Corr* | | |
| subject (Intercept) | 6.77 | 2.60 |  |  |  |
| subject (response type × stimulation site × TMS timing × trial number) | 8.41 | 2.90 | -0.29 |  |  |
| Residual | 36.82 | 6.07 |  |  |  |
| Model fit | | | | | |
|  | marginal | | conditional | | |
| R^2^ | 0.01 | | 0.17 | | |
| Key: *p*-values for fixed effects calculated using Satterthwaites approximations. Model equation: Pe ~ 1 + response type*stimulation site*TMS timing*trial number + (1 + response type:stimulation site:TMS timing:trial number \| subject) | | | | | |
| *Note*. *n*_subjects_ = 24, *n*_observations_ = 7891. Bold text indicates significant effects. | | | | | |
| Supplementary Table S3: Inferential statistics for the LME analysis examining the effect of response type, feedback delay, group, and trial number on ERN amplitudes in the patient study | | | | | |
| Fixed Effects | | | | | |
|  | *Est*/β | *SE* | *df* | *t* | *p* |
| **(Intercept)** | **-1.82** | **0.22** | **49.46** | **-8.32** | **< .001** |
| response type | 0.14 | 0.10 | 15986.61 | 1.38 | .168 |
| feedback delay | 0.08 | 0.09 | 15955.01 | 0.87 | .386 |
| group | 0.50 | 0.44 | 49.46 | 1.14 | .261 |
| trial number | 0.06 | 0.05 | 15957.83 | 1.27 | .205 |
| response type × feedback delay | 0.28 | 0.19 | 15978.81 | 1.48 | .139 |
| response type × group | -0.19 | 0.20 | 15986.61 | -0.95 | .342 |
| **feedback delay × group** | **0.52** | **0.19** | **15955.01** | **2.74** | **.006** |
| response type × trial number | -0.11 | 0.09 | 15975.92 | -1.12 | .263 |
| feedback delay × trial number | 0.04 | 0.09 | 15954.07 | 0.47 | .641 |
| group × trial number | 0.06 | 0.09 | 15957.83 | 0.65 | .518 |
| response type × feedback delay × group | -0.23 | 0.38 | 15978.81 | -0.59 | .554 |
| response type × feedback delay × trial number | -0.36 | 0.19 | 15965.10 | -1.90 | .057 |
| response type × group × trial number | -0.26 | 0.19 | 15975.92 | -1.37 | .170 |
| feedback delay × group × trial number | -0.11 | 0.19 | 15954.07 | -0.57 | .566 |
| response type × feedback delay × group × trial number | -0.64 | 0.38 | 15965.10 | -1.69 | .091 |
| Random Effects | | | | | |
|  | *Variance* | *SD* |  | | |
| subject (Intercept) | 2.31 | 1.52 |  |  |  |
| Residual | 31.66 | 5.63 |  |  |  |
| Model fit | | | | | |
|  | marginal | | conditional | | |
| R^2^ | 0.00 | | 0.07 | | |
| Key: *p*-values for fixed effects calculated using Satterthwaites approximations. Model equation: ERN ~ 1 + response type*feedback delay*group*trial number + (1 \| subject) | | | | | |
| *Note*. *n*_subjects_ = 51, *n*_observations_ = 16014. Bold text indicates significant effects. | | | | | |

| Supplementary Table S4: Inferential statistics for the LME analysis examining the effect of response type, feedback delay, group, and trial number on Pe amplitudes in the patient study | | | | | | | | | | | | | | | | | | |
| --- | --- | --- | --- | --- | --- | --- | --- | --- | --- | --- | --- | --- | --- | --- | --- | --- | --- | --- |
| Fixed Effects | | | | | | | | | | | | | | | | | | |
|  | | *Est*/β | | | | | | *SE* | | | *df* | | | | *t* | | | *p* |
| **(Intercept)** | | **0.86** | | | | | | **0.23** | | | **45.39** | | | | **3.68** | | | **.001** |
| **response type** | | **-0.53** | | | | | | **0.10** | | | **14737.43** | | | | **-5.40** | | | **< .001** |
| **feedback delay** | | **1.02** | | | | | | **0.09** | | | **14197.69** | | | | **10.83** | | | **< .001** |
| group | | -0.18 | | | | | | 0.47 | | | 45.39 | | | | -0.39 | | | .696 |
| **trial number** | | **-0.26** | | | | | | **0.05** | | | **13716.24** | | | | **-5.63** | | | **< .001** |
| response type × feedback delay | | -0.02 | | | | | | 0.19 | | | 14717.76 | | | | -0.10 | | | .921 |
| response type × group | | 0.22 | | | | | | 0.20 | | | 14737.43 | | | | 1.10 | | | .271 |
| feedback delay × group | | 0.02 | | | | | | 0.19 | | | 14197.69 | | | | 0.09 | | | .928 |
| response type × trial number | | 0.08 | | | | | | 0.09 | | | 14680.50 | | | | 0.80 | | | .424 |
| feedback delay × trial number | | 0.06 | | | | | | 0.09 | | | 6743.09 | | | | 0.61 | | | .543 |
| **group × trial number** | | **0.23** | | | | | | **0.09** | | | **13716.24** | | | | **2.48** | | | **.013** |
| response type × feedback delay × group | | 0.24 | | | | | | 0.38 | | | 14717.76 | | | | 0.62 | | | .537 |
| response type × feedback delay × trial number | | -0.03 | | | | | | 0.22 | | | 51.33 | | | | -0.14 | | | .893 |
| response type × group × trial number | | 0.01 | | | | | | 0.19 | | | 14680.50 | | | | 0.08 | | | .937 |
| feedback delay × group × trial number | | -0.06 | | | | | | 0.19 | | | 6743.09 | | | | -0.31 | | | .755 |
| response type × feedback delay × group × trial number | | -0.14 | | | | | | 0.44 | | | 51.33 | | | | -0.31 | | | .756 |
| Random Effects | | | | | | | | | | | | | | | | | | |
|  | | *Variance* | | | | | | *SD* | | |  | | | | | | | |
| subject (Intercept) | | 2.45 | | | | | | 1.56 | | |  | | | |  | | |  |
| subject (response type × feedback delay × group × trial number) | | 0.66 | | | | | | 0.81 | | | -0.14 | | | |  | | |  |
| Residual | | 29.12 | | | | | | 5.40 | | |  | | | |  | | |  |
| Model fit | | | | | | | | | | | | | | | | | | |
|  | | marginal | | | | | | | | | conditional | | | | | | | |
| R^2^ | | 0.01 | | | | | | | | | 0.09 | | | | | | | |
| Key: *p*-values for fixed effects calculated using Satterthwaites approximations. Model equation: Pe ~ 1 + response type*feedback delay*group*trial number + (1 + response type × feedback delay × group × trial number \| subject) | | | | | | | | | | | | | | | | | | |
| *Note*. *n*_subjects_ = 47, *n*_observations_ = 14758. Bold text indicates significant effects. | | | | | | | | | | | | | | | | | | |
| Supplementary Table S5: Inferential statistics for the LME analysis examining the effect of Q_diff_, stimulation site, TMS timing, and trial number on ERN amplitudes in the TMS study | | | | | | | | | | | | | | | | | |  |
| Fixed Effects | | | | | | | | | | | | | | | | | |  |
|  | *Est*/β | | | | *SE* | | | *df* | | | *t* | | | | *p* | | |  |
| **(Intercept)** | **-2.54** | | | | **0.35** | | | **22.98** | | | **-7.25** | | | | **< .001** | | |  |
| **Q_diff_** | **0.43** | | | | **0.08** | | | **4725.26** | | | **5.16** | | | | **< .001** | | |  |
| stimulation site | 0.24 | | | | 0.15 | | | 7234.47 | | | 1.54 | | | | .124 | | |  |
| TMS timing | -0.13 | | | | 0.15 | | | 7235.41 | | | -0.83 | | | | .406 | | |  |
| trial number | -0.08 | | | | 0.08 | | | 5835.77 | | | -1.08 | | | | .283 | | |  |
| Q_diff_ × stimulation site | -0.31 | | | | 0.16 | | | 6268.64 | | | -1.88 | | | | .060 | | |  |
| **Q_diff_ × TMS timing** | **0.52** | | | | **0.16** | | | **7163.28** | | | **3.23** | | | | **.001** | | |  |
| stimulation site × TMS timing | -0.33 | | | | 0.31 | | | 7017.29 | | | -1.06 | | | | .290 | | |  |
| **Q_diff_ × trial number** | **0.24** | | | | **0.08** | | | **958.20** | | | **2.84** | | | | **.005** | | |  |
| stimulation site × trial number | -0.14 | | | | 0.15 | | | 5802.99 | | | -0.88 | | | | .380 | | |  |
| TMS timing × trial number | -0.11 | | | | 0.15 | | | 5917.21 | | | -0.73 | | | | .467 | | |  |
| Q_diff_ × stimulation site × TMS timing | -0.17 | | | | 0.33 | | | 6631.53 | | | -0.52 | | | | .601 | | |  |
| **Q_diff_ × stimulation site × trial number** | **-0.40** | | | | **0.17** | | | **984.23** | | | **-2.44** | | | | **.015** | | |  |
| Q_diff_ × TMS timing × trial number | 0.32 | | | | 0.17 | | | 807.11 | | | 1.92 | | | | .055 | | |  |
| stimulation site × TMS timing × trial number | 0.18 | | | | 0.31 | | | 3875.15 | | | 0.59 | | | | .558 | | |  |
| Q_diff_ × stimulation site × TMS timing × trial number | -0.03 | | | | 0.39 | | | 24.22 | | | -0.08 | | | | .937 | | |  |
| Random Effects | | | | | | | | | | | | | | | | | |  |
|  | *Variance* | | | | *SD* | | | *Corr* | | | | | | | | | |  |
| subject (Intercept) | 2.80 | | | | 1.67 | | |  | | |  | | | |  | | |  |
| subject (Q_diff_ × stimulation site × TMS timing × trial number) | 1.00 | | | | 1.00 | | | -0.01 | | |  | | | |  | | |  |
| Residual | 42.67 | | | | 6.53 | | |  | | |  | | | |  | | |  |
| Model fit | | | | | | | | | | | | | | | | | |  |
|  | marginal | | | | | | | conditional | | | | | | | | | |  |
| R^2^ | 0.01 | | | | | | | 0.07 | | | | | | | | | |  |
| Key: *p*-values for fixed effects calculated using Satterthwaites approximations. Model equation: ERN ~ 1 + Q_diff_*stimulation site*TMS timing*trial number + (1 + Q_diff_:stimulation site:TMS timing:trial number \| subject) | | | | | | | | | | | | | | | | | |  |
| *Note*. *n*_subjects_ = 24, *n*_observations_ = 7479. Bold text indicates significant effects. | | | | | | | | | | | | | | | | | |  |
| Supplementary Table S6: Inferential statistics for the LME analysis examining the effect of Q_diff_, stimulation site TMS timing, and trial number on Pe amplitudes in the TMS study | | | | | | | | | | | | | | |  |  |  |  |
| Fixed Effects | | | | | | | | | | | | | | |  |  |  |  |
|  | *Est*/β | *SE* | | | | | *df* | | | | *t* | *p* | | |  |  |  |  |
| **(Intercept)** | **-1.54** | **0.52** | | | | | **21.98** | | | | **-2.94** | **.008** | | |  |  |  |  |
| **Q_diff_** | **-0.56** | **0.08** | | | | | **5816.27** | | | | **-7.16** | **< .001** | | |  |  |  |  |
| stimulation site | -0.12 | 0.14 | | | | | 7358.48 | | | | -0.88 | .379 | | |  |  |  |  |
| **TMS timing** | **0.60** | **0.14** | | | | | **7347.09** | | | | **4.23** | **< .001** | | |  |  |  |  |
| **trial number** | **0.24** | **0.07** | | | | | **6325.84** | | | | **3.43** | **.001** | | |  |  |  |  |
| **Q_diff_ × stimulation site** | **-0.42** | **0.15** | | | | | **6808.80** | | | | **-2.70** | **.007** | | |  |  |  |  |
| **Q_diff_ × TMS timing** | **0.40** | **0.15** | | | | | **7323.77** | | | | **2.64** | **.008** | | |  |  |  |  |
| **stimulation site × TMS timing** | **-1.04** | **0.28** | | | | | **7250.07** | | | | **-3.70** | **< .001** | | |  |  |  |  |
| Q_diff_ × trial number | 0.01 | 0.08 | | | | | 1640.04 | | | | 0.15 | .885 | | |  |  |  |  |
| stimulation site × trial number | 0.14 | 0.14 | | | | | 6474.92 | | | | 1.02 | .309 | | |  |  |  |  |
| TMS timing × trial number | 0.04 | 0.14 | | | | | 6159.36 | | | | 0.30 | .763 | | |  |  |  |  |
| **Q_diff_ × stimulation site × TMS timing** | **1.30** | **0.31** | | | | | **6933.62** | | | | **4.24** | **< .001** | | |  |  |  |  |
| Q_diff_ × stimulation site × trial number | 0.16 | 0.16 | | | | | 1730.99 | | | | 1.03 | .302 | | |  |  |  |  |
| Q_diff_ × TMS timing × trial number | 0.16 | 0.16 | | | | | 1215.63 | | | | 0.98 | .330 | | |  |  |  |  |
| stimulation site × TMS timing × trial number | 0.23 | 0.29 | | | | | 4212.81 | | | | 0.79 | .429 | | |  |  |  |  |
| Q_diff_ × stimulation site × TMS timing × trial number | 0.43 | 0.45 | | | | | 15.83 | | | | 0.95 | .357 | | |  |  |  |  |
| Random Effects | | | | | | | | | | | | | | |  |  |  |  |
|  | *Variance* | *SD* | | | | | *Corr* | | | | | | | |  |  |  |  |
| subject (Intercept) | 6.20 | 2.49 | | | | |  | | | |  |  | | |  |  |  |  |
| subject (Q_diff_ × stimulation site × TMS timing × trial number) | 2.36 | 1.54 | | | | | -0.31 | | | |  |  | | |  |  |  |  |
| Residual | 36.28 | 6.02 | | | | |  | | | |  |  | | |  |  |  |  |
| Model fit | | | | | | | | | | | | | | |  |  |  |  |
|  | marginal | | | | | | conditional | | | | | | | |  |  |  |  |
| R^2^ | 0.02 | | | | | | 0.16 | | | | | | | |  |  |  |  |
| Key: *p*-values for fixed effects calculated using Satterthwaites approximations. Model equation: Pe ~ 1 + Q_diff_*stimulation site*TMS timing*trial number + (1 + Q_diff_:stimulation site:TMS timing:trial number \| subject) | | | | | | | | | | | | | | |  |  |  |  |
| *Note*. *n*_subjects_ = 23, *n*_observations_ = 7564. Bold text indicates significant effects. | | | | | | | | | | | | | | |  |  |  |  |
| Supplementary Table S7: Inferential statistics for the LME analysis examining the effect of Q_diff_, feedback delay, group, and trial number on ERN amplitudes in the patient study | | | | | | | | | | | | | | | | | | |
| Fixed Effects | | | | | | | | | | | | | | | | | | |
|  | | | | *Est*/β | | | *SE* | | | *df* | | | *t* | | | | *p* | |
| **(Intercept)** | | | | **-1.80** | | | **0.22** | | | **49.01** | | | **-8.36** | | | | **< .001** | |
| Q_diff_ | | | | -0.02 | | | 0.05 | | | 7139.12 | | | -0.40 | | | | .691 | |
| feedback delay | | | | 0.14 | | | 0.09 | | | 13665.62 | | | 1.51 | | | | .131 | |
| group | | | | 0.46 | | | 0.43 | | | 49.01 | | | 1.07 | | | | .290 | |
| trial number | | | | 0.05 | | | 0.05 | | | 9925.32 | | | 1.12 | | | | .264 | |
| Q_diff_ × feedback delay | | | | 0.00 | | | 0.09 | | | 11988.66 | | | -0.01 | | | | .991 | |
| Q_diff_ × group | | | | -0.19 | | | 0.11 | | | 7139.12 | | | -1.83 | | | | .067 | |
| **feedback delay × group** | | | | **0.45** | | | **0.18** | | | **13665.62** | | | **2.49** | | | | **.013** | |
| Q_diff_ × trial number | | | | -0.03 | | | 0.05 | | | 2689.70 | | | -0.68 | | | | .498 | |
| feedback delay × trial number | | | | 0.02 | | | 0.09 | | | 1006.30 | | | 0.27 | | | | .790 | |
| group × trial number | | | | 0.04 | | | 0.09 | | | 9925.32 | | | 0.47 | | | | .642 | |
| **Q_diff_ × feedback delay × group** | | | | **-0.46** | | | **0.19** | | | **11988.66** | | | **-2.46** | | | | **.014** | |
| Q_diff_ × feedback delay × trial number | | | | -0.13 | | | 0.10 | | | 30.84 | | | -1.29 | | | | .207 | |
| Q_diff_ × group × trial number | | | | 0.04 | | | 0.09 | | | 2689.70 | | | 0.39 | | | | .694 | |
| feedback delay × group × trial number | | | | -0.13 | | | 0.18 | | | 1006.30 | | | -0.73 | | | | .465 | |
| Q_diff_ × feedback delay × group × trial number | | | | -0.05 | | | 0.21 | | | 30.84 | | | -0.24 | | | | .809 | |
| Random Effects | | | | | | | | | | | | | | | | | | |
|  | | | | *Variance* | | | *SD* | | | *Corr* | | | | | | | | |
| subject (Intercept) | | | | 2.27 | | | 1.51 | | |  | | |  | | | |  | |
| subject (Q_diff_ × feedback delay × group × trial number) | | | | 0.09 | | | 0.30 | | | 0.76 | | |  | | | |  | |
| Residual | | | | 31.65 | | | 5.63 | | |  | | |  | | | |  | |
| Model fit | | | | | | | | | | | | | | | | | | |
|  | | | | marginal | | | | | | conditional | | | | | | | | |
| R^2^ | | | | 0.00 | | | | | | 0.07 | | | | | | | | |
| Key: *p*-values for fixed effects calculated using Satterthwaites approximations. Model equation: ERN ~ 1 + Q_diff_*feedback delay*group*trial number + (1 + Q_diff_ × feedback delay × group × trial number \| subject) | | | | | | | | | | | | | | | | | | |
| *Note*. *n*_subjects_ = 51, *n*_observations_ = 16014. Bold text indicates significant effects. | | | | | | | | | | | | | | | | | | |
| Supplementary Table S8: Inferential statistics for the LME analysis examining the effect of Q_diff_, feedback delay, group, and trial number on Pe amplitudes in the patient study | | | | | | | | | | | | | | | | | | |
| Fixed Effects | | | | | | | | | | | | | | | | | | |
|  | | | *Est*/β | | | *SE* | | | *df* | | | | | *t* | | *p* | | |
| **(Intercept)** | | | **0.59** | | | **0.25** | | | **39.09** | | | | | **2.41** | | **.021** | | |
| **Q_diff_** | | | **-0.49** | | | **0.06** | | | **7728.01** | | | | | **-7.56** | | **< .001** | | |
| **feedback delay** | | | **1.13** | | | **0.10** | | | **11258.39** | | | | | **11.39** | | **< .001** | | |
| group | | | -0.18 | | | 0.49 | | | 39.09 | | | | | -0.36 | | .722 | | |
| **trial number** | | | **-0.27** | | | **0.05** | | | **6318.58** | | | | | **-5.45** | | **< .001** | | |
| Q_diff_ × feedback delay | | | -0.02 | | | 0.11 | | | 11319.05 | | | | | -0.22 | | .827 | | |
| Q_diff_ × group | | | 0.15 | | | 0.13 | | | 7728.01 | | | | | 1.17 | | .242 | | |
| feedback delay × group | | | -0.12 | | | 0.20 | | | 11258.39 | | | | | -0.60 | | .548 | | |
| **Q_diff_ × trial number** | | | **-0.12** | | | **0.06** | | | **2877.32** | | | | | **-2.12** | | **.034** | | |
| feedback delay × trial number | | | 0.08 | | | 0.10 | | | 729.06 | | | | | 0.82 | | .413 | | |
| **group × trial number** | | | **0.32** | | | **0.10** | | | **6318.58** | | | | | **3.25** | | **.001** | | |
| **Q_diff_ × feedback delay × group** | | | **-0.58** | | | **0.23** | | | **11319.05** | | | | | **-2.54** | | **.011** | | |
| Q_diff_ × feedback delay × trial number | | | 0.06 | | | 0.14 | | | 32.89 | | | | | 0.48 | | .637 | | |
| Q_diff_ × group × trial number | | | 0.14 | | | 0.11 | | | 2877.32 | | | | | 1.23 | | .220 | | |
| feedback delay × group × trial number | | | -0.04 | | | 0.21 | | | 729.06 | | | | | -0.21 | | .834 | | |
| Q_diff_ × feedback delay × group × trial number | | | -0.16 | | | 0.27 | | | 32.89 | | | | | -0.60 | | .553 | | |
| Random Effects | | | | | | | | | | | | | | | | | | |
|  | | | *Variance* | | | *SD* | | | *Corr* | | | | | | | | | |
| subject (Intercept) | | | 2.37 | | | 1.54 | | |  | | | | |  | |  | | |
| subject (Q_diff_ × feedback delay × group × trial number) | | | 0.21 | | | 0.45 | | | 0.03 | | | | |  | |  | | |
| Residual | | | 29.46 | | | 5.43 | | |  | | | | |  | |  | | |
| Model fit | | | | | | | | | | | | | | | | | | |
|  | | | marginal | | | | | | conditional | | | | | | | | | |
| R^2^ | | | 0.02 | | | | | | 0.09 | | | | | | | | | |
| Key: *p*-values for fixed effects calculated using Satterthwaites approximations. Model equation: Pe ~ 1 + Q_diff_*feedback delay*group*trial number + (1 + Q_diff_ × feedback delay × group × trial number \| subject) | | | | | | | | | | | | | | | | | | |
| *Note*. *n*_subjects_ = 41, *n*_observations_ = 12893. Bold text indicates significant effects. | | | | | | | | | | | | | | | | | | |

References

1. Huvermann D, Berlijn A, Thieme A, Erdlenbruch F, Groiss SJ, Deistung A, et al. The cerebellum contributes to prediction error coding in reinforcement learning in humans. J Neurosci. 2025;e1972242025.

2. Oldfield RC. The assessment and analysis of handedness: The Edinburgh inventory. Neuropsychologia. 1971 Mar 1;9(1):97–113.

3. Merz J, Lehrl S, Galster V, Erzigkeit H. MWT-B—Ein Intelligenzkurztest. Psychiatr Neurol Med Psychol (Leipz). 1975;27(7):423–8.

4. Beck AT, Steer RA, Brown G. Beck depression inventory–II. Psychol Assess. 1996;

5. Schmitz-Hübsch T, Tezenas du Montcel, S., Baliko L, Berciano J, Boesch S, C. Depondt, et al. Scale for the assessment and rating of ataxia. Neurology. 2006 Jun 13;66(11):1717.

6. Chatrian GE, Lettich E, Nelson PL. Ten percent electrode system for topographic studies of spontaneous and evoked EEG activities. Am J EEG Technol. 1985 Jun 1;25(2):83–92.

7. Sutton RS, Barto AG. Temporal-Difference Learning. In: Reinforcement Learning: an introduction. 2nd ed. Cambridge, MA: The MIT press; 2018. p. 119–40.

8. Bray S, O’Doherty J. Neural Coding of Reward-Prediction Error Signals During Classical Conditioning With Attractive Faces. J Neurophysiol. 2007 Apr 1;97(4):3036–45.

9. Chase HW, Swainson R, Durham L, Benham L, Cools R. Feedback-related negativity codes prediction error but not behavioral adjustment during probabilistic reversal learning. J Cogn Neurosci. 2011 Apr 1;23(4):936–46.

10. Ichikawa N, Siegle GJ, Dombrovski A, Ohira H. Subjective and model-estimated reward prediction: Association with the feedback-related negativity (FRN) and reward prediction error in a reinforcement learning task. Int J Psychophysiol. 2010 Dec 1;78(3):273–83.

11. McDougle SD, Butcher PA, Parvin DE, Mushtaq F, Niv Y, Ivry RB, et al. Neural signatures of prediction errors in a decision-making task are modulated by action execution failures. Curr Biol. 2019 May 20;29(10):1606-1613.e5.

12. Murray GK, Corlett PR, Clark L, Pessiglione M, Blackwell AD, Honey G, et al. Substantia nigra/ventral tegmental reward prediction error disruption in psychosis. Mol Psychiatry. 2008 Mar 1;13(3):267–76.

13. Pessiglione M, Seymour B, Flandin G, Dolan RJ, Frith CD. Dopamine-dependent prediction errors underpin reward-seeking behaviour in humans. Nature. 2006 Aug 1;442(7106):1042–5.

14. Katahira K, Yuki S, Okanoya K. Model-based estimation of subjective values using choice tasks with probabilistic feedback. J Math Psychol. 2017 Aug 1;79:29–43.
